# Allostatic Self-efficacy: A Metacognitive Theory of Dyshomeostasis-Induced Fatigue and Depression

**DOI:** 10.3389/fnhum.2016.00550

**Published:** 2016-11-15

**Authors:** Klaas E. Stephan, Zina M. Manjaly, Christoph D. Mathys, Lilian A. E. Weber, Saee Paliwal, Tim Gard, Marc Tittgemeyer, Stephen M. Fleming, Helene Haker, Anil K. Seth, Frederike H. Petzschner

**Affiliations:** ^1^Translational Neuromodeling Unit, Institute for Biomedical Engineering, University of Zurich and ETH ZurichZurich, Switzerland; ^2^Wellcome Trust Centre for Neuroimaging, University College LondonLondon, UK; ^3^Max Planck Institute for Metabolism ResearchCologne, Germany; ^4^Department of Neurology, Schulthess ClinicZurich, Switzerland; ^5^Center for Complementary and Integrative Medicine, University Hospital ZurichZurich, Switzerland; ^6^Sackler Centre for Consciousness Science, School of Engineering and Informatics, University of SussexBrighton, UK

**Keywords:** computational psychiatry, effective connectivity, dynamic causal modeling, homeostasis, allostasis, predictive coding, active inference, multiple sclerosis

## Abstract

This paper outlines a hierarchical Bayesian framework for interoception, homeostatic/allostatic control, and meta-cognition that connects fatigue and depression to the experience of chronic dyshomeostasis. Specifically, viewing interoception as the inversion of a generative model of viscerosensory inputs allows for a formal definition of dyshomeostasis (as chronically enhanced surprise about bodily signals, or, equivalently, low evidence for the brain's model of bodily states) and allostasis (as a change in prior beliefs or predictions which define setpoints for homeostatic reflex arcs). Critically, we propose that the performance of interoceptive-allostatic circuitry is monitored by a metacognitive layer that updates beliefs about the brain's capacity to successfully regulate bodily states (allostatic self-efficacy). In this framework, fatigue and depression can be understood as sequential responses to the interoceptive experience of dyshomeostasis and the ensuing metacognitive diagnosis of low allostatic self-efficacy. While fatigue might represent an early response with adaptive value (cf. sickness behavior), the experience of chronic dyshomeostasis may trigger a generalized belief of low self-efficacy and lack of control (cf. learned helplessness), resulting in depression. This perspective implies alternative pathophysiological mechanisms that are reflected by differential abnormalities in the effective connectivity of circuits for interoception and allostasis. We discuss suitably extended models of effective connectivity that could distinguish these connectivity patterns in individual patients and may help inform differential diagnosis of fatigue and depression in the future.

## Introduction

Fatigue is a prominent symptom of major clinical significance, not only in chronic fatigue syndrome (CFS) *per se*, but across a wide range of immunological and endocrine disorders, cancer and neuropsychiatric diseases (for overviews, see Wessely, 2001; Chaudhuri and Behan, 2004; Dantzer et al., 2014). For example, it is the most frequent (Stuke et al., 2009) symptom in Multiple Sclerosis (MS), with major impact on quality of life. It is strongly associated with depression (Wessely et al., 1996; Bakshi et al., 2000; Kroencke et al., 2000; Pittion-Vouyovitch et al., 2006), and longitudinal studies have demonstrated that fatigue represents a risk factor for depression (and vice versa; Skapinakis et al., 2004). Additionally, fatigue represents a core criterion for the diagnosis of major depression in standard psychiatric classification schemes (ICD-10 and DSM-5).

The clinical concept of fatigue is a heterogeneous construct, comprising at least two dimensions (Kluger et al., 2013): fatiguability of cognitive and motor processes, and subjective perception of fatigue. While the former can be measured objectively, the latter requires self-report via questionnaires. Given its clinical importance, it has been remarkably difficult to develop a theory of fatigue that is comprehensive, specific and allows for developing objective clinical tests (Wessely, 2001). Research on its pathophysiology has largely focused on molecular processes, particularly in the context of inflammation (Dantzer et al., 2014; Patejdl et al., 2016), but efforts to link these molecular processes to the physiology and computation (information processing) of cerebral circuits are rare. This paper attempts to address this challenge and outlines the foundations of a theory of fatigue that is grounded in interoception (Craig, 2002) and homeostatic/allostatic control (Sterling, 2012), offering a formal (hierarchical Bayesian) perspective on (some of) the computations involved. In particular, we propose a metacognitive mechanism that explains the sequential occurrence of fatigue and depression, given a state of prolonged dyshomeostasis.

This paper has the following structure. First, we discuss why disease theories of fatigue confined to the molecular/cellular level are not sufficient for a comprehensive understanding of fatigue, but need to be complemented by a computational perspective. Second, as a basis for developing this perspective, we review long-standing notions from systems theory and control theory and their implications for interoception as well as homeostatic and allostatic control. Third, we apply a hierarchical Bayesian view to fatigue and cast it as a meta-cognitive phenomenon: a belief of failure at one's most fundamental task—homeostatic/allostatic regulation—which arises from experiencing enhanced interoceptive surprise. We suggest that fatigue is a (possibly adaptive) initial allostatic response to a state of interoceptive surprise; if dyshomeostasis continues, the belief of low allostatic self-efficacy and lack of control may pervade all domains of cognition and manifests as a generalized sense of helplessness, with depression as a consequence. Fourth, we derive specific predictions against which this theory can be tested and outline the necessary methodological extensions of contemporary models of effective connectivity, such as DCM. Finally, we consider how such extended generative models might become useful for differential diagnosis of fatigue in the future.

## The need for a computational theory of fatigue

Existing pathophysiological theories of fatigue mainly refer to inflammatory and metabolic processes at the molecular level. For example, a longstanding observation is that pro-inflammatory cytokines, resulting from peripheral (extra-cerebral) immunological processes, induce “sickness behavior” (Dantzer and Kelley, 2007) with fatigue as a key symptom. This may result from a range of different mechanisms, including reduced synthesis of monoaminergic transmitters or inflammation-induced shifts in the production of metabolites such as kynurenines, which impact on transmission at glutamatergic synapses (for a comprehensive recent review, see Dantzer et al., 2014).

While these hypotheses have been very influential and useful in suggesting potential future treatment avenues, they do not, on their own, allow for constructing a comprehensive theory of fatigue. First, as for any neuropsychiatric symptom, we eventually need a theory that unifies and links disease processes across molecular, cellular and circuit (systems) levels of description. This is important because a theory of fatigue that is confined to the molecular level does not explain how clinical symptoms arise; by contrast, a circuit-level description is the closest we can presently get to behavior and subjective experience. Moreover, neuropsychiatric disease processes can not only originate from the molecular level and spread “bottom-up,” causing cellular and circuit-level disturbances; in addition, the reverse (top-down) direction and the ubiquitous existence of reciprocal brain-body interactions are well-established (Sapolsky, 2015). For example, seemingly maladaptive behavior can materialize as the (optimal) consequence of beliefs that form under exposure to specific environmental input statistics (Schwartenbeck et al., 2015b). That is, in the absence of any primary molecular or synaptic pathology, exposure to unusual environmental events can induce distorted beliefs about the causal structure of the world, e.g., that it is inherently unpredictable or uncontrollable (cf. learned helplessness; Abramson et al., 1978). Such beliefs engender misdirected coping behavior and have profound physiological consequences, including a dysregulation of cerebral control over endocrine and autonomic nervous system processes (e.g., aberrant activation of the hypothalamic-pituitary axis; HPA; Tsigos and Chrousos, 2002). Importantly, the ensuing immunological and metabolic disturbances in the body exert strong feedback effects on cerebral circuits. For example, stress-related increases in levels of cortisol and pro-inflammatory cytokines affect NMDA receptor (NMDAR) function (Nair and Bonneau, 2006; Gruol, 2015; Vezzani and Viviani, 2015). Importantly, NMDAR dependent signaling is thought to be essential for updating and encoding representations of beliefs (Corlett et al., 2010; Vinckier et al., 2016). This suggests that peripheral inflammatory or endocrine disturbances could impede the adjustment of aberrant beliefs by which they were caused in the first place (i.e., a positive feedback loop). In brief, the existence of closed-loop interactions between cognitive and bodily processes implies that we require a wider theory of fatigue than one focusing on molecular events alone.

**Figure 1:**
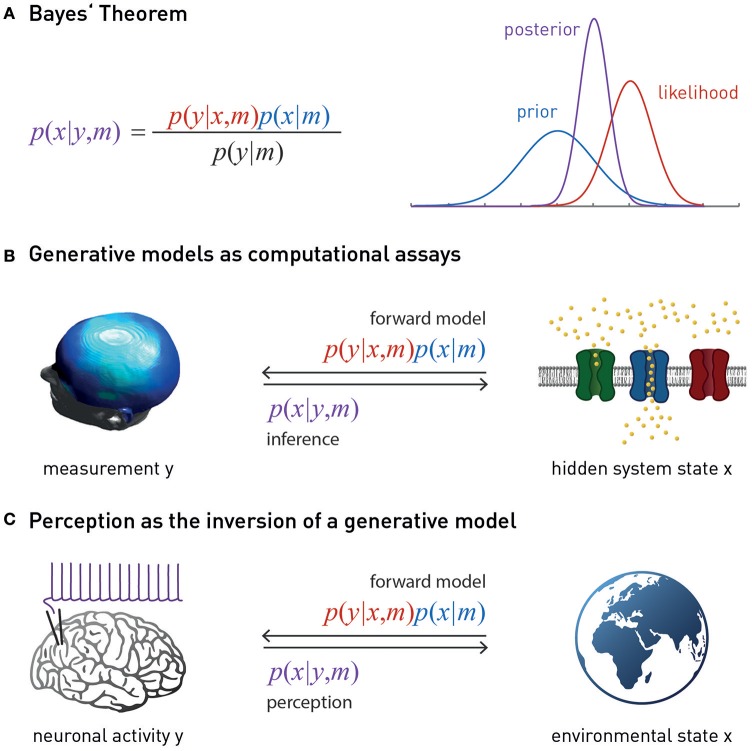
**(A)** Bayes theorem provides the foundation for a generative model *m*. This combines the likelihood function *p*(*y* | *x, m*) (a probabilistic mapping from hidden states of the world, *x*, to sensory inputs *y*) with the prior *p*(*x* | *m*) (an *a priori* probability distribution of the world's states). Model inversion corresponds to computing the posterior *p*(*x* | *y, m*), i.e., the probability of the hidden states, given the observed data *y*. The posterior is a “compromise” between likelihood and prior, weighted by their relative precisions. The model evidence *p*(*y* | *m*) in the denominator of Bayes' theorem is a normalization constant that forms the basis for Bayesian model comparison—see main text. **(B)** Suitably specified and validated generative models with mechanistic (e.g., physiological or algorithmic) interpretability could be used as a computational assay for diagnostic purposes. The left graphics is reproduced, with permission, from Garrido et al. (2008). **(C)** Contemporary models of perception (the “Bayesian brain hypothesis”) assume that the brain instantiates a generative model of its sensory inputs. Perception corresponds to inverting this model, yielding posterior beliefs about the causes of sensory inputs. The globe picture is freely available from http://www.vectortemplates.com/raster-globes.php.

There is a second, more practical, reason why a purely molecular/cellular theory of fatigue cannot provide a clinically sufficient account of fatigue: molecular disease processes in brain tissue are not easily accessible for non-invasive diagnostics in humans. The relative separation of the brain from the body by the blood-brain barrier means that we only have indirect access to brain tissue, such as biochemical analyses of cerebrospinal fluid (CSF), and there are very few diagnostic questions (e.g., in neuro-oncology or epilepsy) where the risks of brain tissue biopsies or invasive recordings are justified by diagnostic benefits. However, provided we have a concrete model that specifies how a disease process at the molecular/cellular level leads to measurable changes in the activity of specific brain circuits, one can, in principle, infer the expression of this process from non-invasive neuroimaging and electrophysiological measurements, such as functional magnetic resonance imaging (fMRI) or magneto-/electroencephalography (M/EEG). Technically, this involves a so-called “generative model” *m* which specifies how the hidden (unobservable) state *x* of a neuronal circuit translates probabilistically into a measurement obtained *y* with fMRI or M/EEG, and which can be used to infer hidden states from measurements (Figure 1). Using a generative model of brain activity or behavioral measurements to address diagnostic questions amounts to a “computational assay” (Stephan and Mathys, 2014; Stephan et al., 2015). The application of generative models to clinical questions is presently beginning to take place across the whole range of neuropsychiatry, including applications to schizophrenia (Schlagenhauf et al., 2014), depression (Hyett et al., 2015), bipolar disorder (Breakspear et al., 2015), Parkinson's disease (Herz et al., 2014), channelopathies (Gilbert et al., 2016), or epilepsy (Cooray et al., 2015). One particular approach we return to below is the generative modeling of neurophysiological circuits. For example, dynamic causal models (DCMs; for reviews see Daunizeau et al., 2011; Friston et al., 2013) allow one to infer directed synaptic connections (effective connectivity) from neuroimaging or electrophysiological data.

Generative modeling is an attractive approach for establishing differential diagnostic procedures. However, given the myriad of possible disease processes, guidance by clinical theories is crucial for the development of computational assays. The framework outlined in this paper is meant to inform the development generative models that infer mechanisms of fatigue and depression from fMRI and MEG/EEG data. The predictions by this framework suggest that differential diagnosis could be decisively facilitated by model-based estimates of directed synaptic connectivity (effective connectivity) within interoceptive circuits and their interactions with regions potentially involved in meta-cognition.

## Teleological brain theories as fundament for understanding fatigue

The diverse behavioral, cognitive and emotional facets of fatigue, its occurrence in numerous syndromatically defined diseases, and the multitude of findings from immunology, neurophysiology and psychology offer a large number of degrees of freedom for “bottom-up” explanations of this complex symptom (Dantzer et al., 2014; Patejdl et al., 2016). Given this complexity, investigating fatigue requires guidance by formal theories which provide top-down constraints on organizing and interpreting the diversity of experimental findings. These top-down constraints could be derived, for example, from theories about the purpose, structure and biophysical implementation of the brain's computations^1^. This strategy is at the heart of an emerging discipline, “Computational Psychiatry” (Montague et al., 2012; Stephan and Mathys, 2014; Friston et al., 2014b; Huys et al., 2016), and has shown promise in tackling other complex neuropsychiatric symptoms, such as delusions (Corlett et al., 2010).

Although its historical roots have rarely been discussed so far, Computational Psychiatry builds on seminal teleological theories of biological (and other) systems that provide fundamental constraints for any attempt of understanding brain function. These include, for example, general systems theory (Von Bertalanffy, 1969), cybernetics and control theory (Wiener, 1948; Ashby, 1954, 1956; Conant and Ashby, 1970; Powers, 1973; Carver and Scheier, 1982; von Foerster, 2003; Seth, 2015a,b,c) and constructivism (Richards and von Glasersfeld, 1979). Some core ideas from these general theories of inference and control in biological systems have laid the foundation for recent concepts of perception and action in computational neuroscience (e.g., Mumford, 1992; Dayan et al., 1995; Rao and Ballard, 1999; Friston, 2005, 2010; Friston et al., 2006; Doya et al., 2011). For example, the central notion of radical constructivism that the brain actively “constructs” a subjective reality from noisy and ambiguous sensory inputs (Richards and von Glasersfeld, 1979; von Foerster, 2003)—as opposed to the brain representing an objective outer reality that is reflected by sensory inputs—are expressed formally, using the language of probability theory, in hierarchical Bayesian models we encounter below. Other central ideas—such as the notion that cognitive systems are self-referential and monitor themselves (von Foerster, 2003) are yet to be exploited fully, e.g., for models of metacognition.

This paper represents a first attempt to use some of these principles for articulating a novel theory of fatigue and how it may transition to depression. In brief, our account views fatigue and depression as metacognitive phenomena: a set of beliefs held by the brain about its own functional capacity—specifically, a perceived lack of control over bodily states. This belief arises when attempts of homeostatic regulation fail to reduce the experience of chronic dyshomeostasis: enduring deviations between expected and sensed bodily states. These persistent deviations or prediction errors signal interoceptive surprise or, equivalently, low evidence of the brain's model of bodily processes. Before we can turn to this notion in more detail, we review some ideas on the role of perception (inference) and prediction (action selection) for homeostasis which originate from the longstanding literature mentioned above and have resurfaced in more recent work in computational neuroscience.

### The brain as an organ for homeostatic control

The brain is literally “embodied”: its structural and functional integrity depends on mechanical support, energy supply, and the provision of a suitable biochemical milieu provided by the body. As a corollary, the selectionist pressures which act upon the brain during evolution cannot be uncoupled from those acting upon the body's *milieu intérieur* (Claude Bernard); i.e., control of bodily homeostasis must constitute a primary purpose of brain function (Cannon, 1929). This control has long been known to involve reflex-like actions (comprising motor, endocrine, immunological, and autonomic processes) that are driven by feedback and the resulting “prediction error”—the discrepancy between an expected bodily state (a homeostatic setpoint^2^) and its actual level as signaled by sensory inputs from the body (Modell et al., 2015); see **Figure 4**. Feedback- or error-based reactive control has been studied for many vitally important variables (such as blood acidity, body temperature, blood levels of glucose and calcium, plasma osmolality) (Woods and Wilson, 2013), and the anatomy and physiology of neuronal circuits involved have been mapped out in detail by physiologists over many decades.

While this reactive type of control dominates the classical literature on homeostasis, it likely only represents the lowest layer in a hierarchy of temporally extended control mechanisms, with most immediate consequences. By contrast, assuming that the brain maintains a model of bodily states and the external environment, higher levels enable prospective control, with two essential components: *inference* (on current bodily state) and *prediction* (of its future evolution, on its own and in response to chosen actions) (Sterling, 2012; Penny and Stephan, 2014; Pezzulo et al., 2015; Seth, 2015a). There are several reasons why homeostatic regulation requires a model that enables inference (perception) and prediction (action selection). First, control is “blind” without perceptual inference: the brain does not have direct access to either bodily or environmental states, but has to infer them from sensory inputs which are inherently noisy and ambiguous. Disentangling the many external states that could underlie any given sensory input is an ill-posed inverse problem that requires constraints or regularization (e.g., by the priors of a generative model; Lee and Mumford, 2003; Kersten et al., 2004). Second, numerous experimental observations indicate that the brain engages in regulatory responses *prior* to a homeostatic perturbation, provided it can be anticipated (Sterling, 2012). In other words, a predicted deviation from a homeostatic setpoint is avoided by choosing suitable actions in advance. Importantly, setpoints are hierarchically structured, and changes in hierarchically lower setpoints may be necessary to prevent departure of bodily state from higher setpoints (Powers, 1973). For example, a temporary change in (lower) setpoints for blood pressure and catecholamine levels may be elicited to engage in fight-flight behavior that is necessary to ensure bodily integrity (higher setpoint). As we will see below, this longstanding notion of hierarchically structured homeostatic setpoints fits nicely to hierarchical Bayesian architectures, where the prior belief at one level is constrained by the prior belief at the next higher level.

This anticipatory control or *allostasis* (“stability through change”; Sterling, 2014) necessarily requires a model capable of generating predictions. The notion of model-based allostatic regulation is a special case of the more general and long-standing view that the brain requires a model of the external world in order to implement optimal control. Specifically, seminal work by Conant and Ashby (1970) has resulted in an influential theorem “[…] which shows, under very broad conditions, that any regulator that is maximally both successful and simple must be isomorphic with the system being regulated. […] The theorem has the interesting corollary that the living brain, so far as it is to be successful and efficient as a regulator for survival, must proceed, in learning, by the formation of a model (or models) of its environment.”

This notion of anticipatory homeostatic control (allostatic control) has important ramifications. Significant perturbations of bodily states arise from the physical and social environments through which the brain navigates the body. For example, basic properties of the physical environment (e.g., ambient temperature, weather, physical activity required by geographical conditions, availability of food and water) have delayed but severe effects on key homeostatic variables (such as body temperature, blood glucose levels, plasma osmolality); these must be predicted in advance and incorporated into the selection of actions in order to avoid fatal effects (for some simple simulations, see Penny and Stephan, 2014). Similarly, in the social domain, learning about the (potentially hostile) intentions of other agents in a reactive way, by trial and error, is risky. Instead, a model or “theory of mind” of other agents' mental states (Frith and Frith, 2012), perhaps grounded in the prolonged interaction with early-life caregivers, is required to predict and avoid interactions with potentially deleterious consequences for social status, access to resources, and ultimately bodily integrity. This means that anticipatory control of bodily states would be drastically incomplete if the brain did not possess a model which enabled inference on current states of the physical and social environment and predicted their trajectories into the future. In brief, principles of anticipatory homeostatic control and the necessity of model-based prediction must generalize beyond the body and apply to physical and social domains of the external world. In the following, the term “external world” is used to refer to both the body and the physical and social world outside the body; this is for notational brevity only and not meant to disregard differences in how bodily, social and physical states can influence brain activity in general and the emergence of fatigue in particular; a topic we return to below.

### Generative models

The notion that the brain maintains and continuously updates a model of its external world for perceptual inference and anticipatory control has been around for a considerable period (Conant and Ashby, 1970). What could such a model look like? Across various proposals, two main design features re-occur and are supported by strong theoretical and empirical arguments. That is, (i) the brain's model is likely to follow principles of probability theory and hence represent a “generative” model; and (ii) structurally, it is plausible to assume that this has a hierarchical structure.

A so-called “generative model” directly follows from the basic laws of probability theory and essentially implements Bayes' theorem—a simple but fundamental statement about how uncertain sources of information (represented by conditional probabilities) can be combined (Figure 1). In the context of perception, a generative model *m* combines a “likelihood function” *p*(*y* | *x, m*) (a probabilistic mapping from hidden states of the world, *x*, to sensory inputs *y*), with a “prior” *p*(*x* | *m*) (an *a priori* probability distribution of the world's states) (Figure 1). The likelihood describes how any given state of the world causes a sensory input with a certain probability; the prior expresses the range of values environmental states inhabit *a priori* and thus encodes learned environmental statistics. One way to understand why this model is called “generative” is to note that it can be used to generate or simulate sensory inputs (data): this simply requires that one samples a value from the prior distribution and plugs it into the likelihood function. This process can be turned around: that is, given some observed data (experienced sensory inputs), Bayes' theorem allows one to compute the probability of the hidden states (the “posterior” *p*(*x* | *y, m*))—this is inference:

(1) $$\begin{array}{r} p\left(x\;|y,m\right)=\frac{p\left(y|x,m\right)p\left(x|m\;\right)}{p\left(y|m\right)} \end{array}$$

As inference corresponds to inverting the process of data generation (from hidden states to sensory inputs), it is also referred to as “inversion” of the generative model, or solving the “inverse problem.” Finally, an important component of a generative model is the model evidence *p*(*y* | *m*) (the denominator from Bayes' theorem). The evidence represents a principled measure of the goodness of a generative model which trades-off accuracy and complexity (Stephan et al., 2009; Penny, 2012); notably, its logarithm relates to the information-theoretic concept of (Shannon) surprise, *S* (sometimes also referred to as surprisal or self-information to distinguish it from psychological notions of surprise). Specifically, the log evidence is identical to negative surprise about seeing the data under model *m*:

(2) $$\begin{array}{r} \log p\left(y|m\right)=-S\left(y|m\right) \end{array}$$

In other words, a good model is one that minimizes the surprise about encountering the data. Conversely, persistent surprise is the hallmark of a bad model.

**Figure 2:**
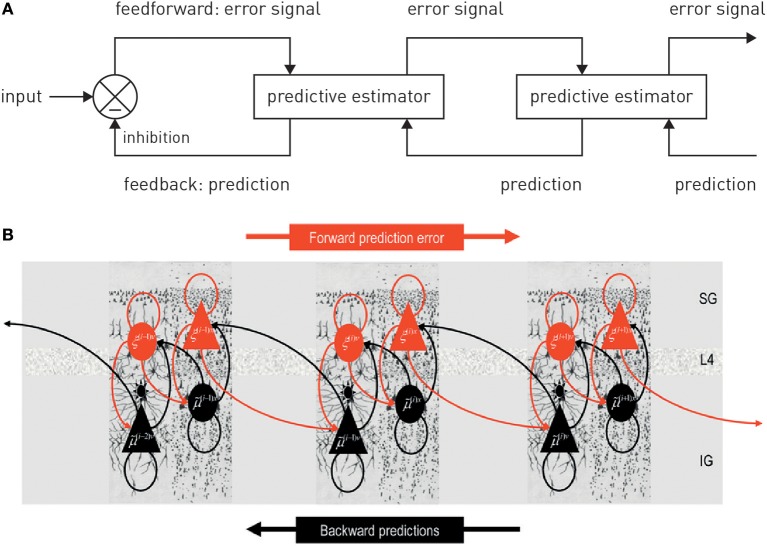
**(A)** A graphical summary of predictive coding. See main text for details. Figure reproduced, with permission, from Rao and Ballard (1999). **(B)** A possible neuronal implementation of predictive coding. See main text for details. Figure reproduced, with permission, from Friston (2008).

**Figure 3:**
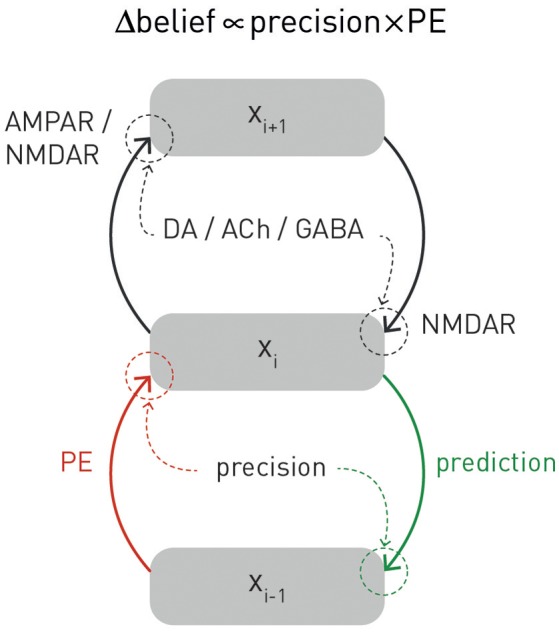
**A graphical summary of computational and physiological key components of hierarchical Bayesian inference**. Computationally, prediction errors are conveyed by ascending or forward connections, while predictions are signaled via backward or descending connections. Critically, both experience a weighting by precision. Physiologically, the currently available evidence suggests that, in cortex, prediction errors are signaled via ionotropic glutamatergic receptors (AMPA and NMDA receptors), predictions via NMDA receptors, while precision-weighting is either implemented through neuromodulatory inputs (e.g., dopamine or acetylcholine) or by local GABAergic mechanisms. The figure is adapted, with permission, from Stephan et al. (2016b).

Generative models can be expressed in a hierarchical form, where each level provides a prediction (prior) for the state of the level below; this prediction can be compared against the actual state (likelihood), resulting in a prediction error which can be signaled upwards for updating the prior (Figures 2, 3). This is an extremely general concept which not only underlies common models in statistics (Kass and Steffey, 1989), but provides a key metaphor for models of brain function (e.g., Rao and Ballard, 1999; Lee and Mumford, 2003; Friston, 2005, 2008; Petzschner et al., 2015), such as predictive coding described below.

### The “Bayesian brain”

The hierarchical form of generative models fits remarkably well to structural principles of cortical organization, where the sensory processing streams consist of hierarchically related cortical areas. This hierarchy is defined anatomically in terms of different cytoarchitectonic properties and types of synaptic connections (bottom-up/ascending/forward connections vs. top-down/descending/backward connections) (Felleman and Van Essen, 1991; Hilgetag et al., 2000). These connections are thought to have different functional properties which are compatible with hierarchical Bayesian inference. For example, in the visual system, anatomical and physiological studies suggest that descending connections convey predictions about activity in lower areas (e.g., Alink et al., 2010; Nassi et al., 2013; Vetter et al., 2015) and have largely inhibitory effects (e.g., Angelucci and Bressloff, 2006; Andolina et al., 2013), as required for “explaining away” in predictive coding (see the discussion in Nassi et al., 2013). Furthermore, pharmacological and computational studies of the auditory mismatch negativity (MMN) system have provided evidence for NMDA receptor dependent signaling of prediction errors via ascending connections (Wacongne et al., 2012; Schmidt et al., 2013). In summary, while definitive proof is outstanding, there is general consensus that ascending connections serve to signal prediction errors up the hierarchy, while predictions are communicated from higher to lower areas via descending connections (for reviews, see Friston, 2005; Corlett et al., 2009; Figure 3 provides an overview).

In the past two decades, theories of perception have converged on the idea that perception corresponds to inverting a hierarchical generative model of sensory inputs (Dayan et al., 1995; Rao and Ballard, 1999; Friston, 2005). In some sense, this idea is not new: more than a century ago, the physiologist Helmholtz already suggested that the brain would have to invert the process of how a visual image was generated in order to infer the underlying physical cause (perception as “unconscious inference”; Helmholtz, 1860/1962). The more recent formalization of this notion under principles of probability theory is commonly referred to as the “Bayesian brain” hypothesis (Friston, 2010; Doya et al., 2011). In addition to the reasons given above, the general idea of perception as inversion of a hierarchical generative model derives from numerous empirical observations and theoretical arguments. Here, we briefly summarize a few central points and point the interested reader to more detailed literature. First, the sensory inputs the brain receives are noisy and often show a non-linear dependence on states in the world; this introduces the need for regularization by prior expectations or knowledge (Friston, 2003; Lee and Mumford, 2003; Kersten et al., 2004). Second, it can be shown that the integration of uncertain sources of information according to principles of probability theory (Bayesian inference) is optimal; this implies that the brain should have evolved to implement perceptual inference in the way such that Bayesian inference is approximated (Geisler and Diehl, 2002). Third, a large body of psychophysical experiments indicate that basic perceptual judgements and multi-sensory integration show clear evidence for the operation of Bayesian inference (for overviews, Knill and Richards, 1996; Geisler and Kersten, 2002; Petzschner et al., 2015). Finally, a generative model not only supports inference, but also allows for predictions. This can be achieved in several ways, for example, predictions about future sensory inputs can be derived from the model's posterior predictive density, and predictions about future states of the world under a chosen action or goal can be derived from the model's posterior dynamics (for example, see Penny and Stephan, 2014).

This link from inference to prediction is important because it provides a basis for coupling perception to action; a fundamental basis for homeostatic control, as described above. Generally, the challenge of control is framed by asking, informed by an estimate of the current state of the world (and possibly a prediction how it evolves), what action optimizes a particular criterion (a “utility function” or “cost function”). One framework to address this challenge is Bayesian decision theory (Körding, 2007; Dayan and Daw, 2008; Daunizeau et al., 2010). In a nutshell, this identifies an optimal action as one that maximizes the “expected utility” (where “expected” refers to a weighted average; i.e., the predicted outcomes are weighted by their relative uncertainty). The definition of utility, however, is not trivial. One common choice is to define utility in relation to “rewards.” This, however, only shifts the problem and raises the question what constitutes “reward” for the brain (compare the discussion in Friston et al., 2012). From a homeostatic perspective, the utility or reward afforded by a particular action depends on four estimates based on inference and prediction:

- an estimate of the current bodily state (interoception);
- an estimate of the current environmental state (exteroception);
- a prediction of how these states would evolve in time (provided by a model of bodily and environmental dynamics);
- and a prediction to what degree the action considered will keep bodily state close to a homeostatic setpoint over time (allostatic control).

Current models of decision-making do not incorporate all of these aspects, and first attempts of accounting for homeostasis and allostasis in formal models of decision-making have only surfaced relatively recently (e.g., Keramati and Gutkin, 2014; Penny and Stephan, 2014; Pezzulo et al., 2015).

Importantly, perception and action do not operate in isolation, nor is there a unidirectional dependency of action on perception. Any chosen action changes the world (and/or the way the brain samples it^3^) and hence the feedback the brain receives in terms of new sensory inputs. This sensory feedback (likelihood function) is combined with the current prior belief (prediction) held by the agent, resulting in a belief update about the state of the world (posterior probability) which, in turn, can inform new actions. This closes the loop from perception to action.

In summary, this section discussed homeostatic and allostatic control as fundamental objectives for the brain and reviewed long-standing concepts that highlight the importance of closed loops of perception and action. In particular, we have emphasized the notion that homeostatic control is not simply reactive, but proactive or anticipatory, and rests on a model of the external world which includes both the body and the influences it may receive from physical and social domains of the environment. Of course, these ideas raise the question how predictive models of this sort may actually be implemented by the brain. This question has been addressed by several recent theories, usually with a focus either on the body (Seth et al., 2011; Seth, 2013, 2015a; Feldman-Barrett and Simmons, 2015) or its environment (Rao and Ballard, 1999; Friston, 2005). In the next section, we review two classes of theories—predictive coding and active inference—which have recently begun to find application to questions of interoception and homeostasis.

### Predictive coding and hierarchical filtering

Predictive coding is a long-standing idea about neural computation that was initially formulated for information processing in the retina (Srinivasan et al., 1982). In its current form, predictive coding postulates that perception rests on the inversion of a hierarchical generative model of sensory inputs which reflects the hierarchical structure of the environment and predicts how sensory inputs are generated from (physical) causes in the world (Rao and Ballard, 1999; Friston, 2005). By inverting this model, the brain can infer the most likely cause (environmental state) underlying sensory input; this process of inference corresponds to perception. At any given level of the model, it is the (precision-weighted, see below) “prediction error” that is of interest—the deviation of the actual input from the expected input. Prediction errors signal that the model needs to be updated and thus drive inference and learning.

Anatomically, models of predictive coding are inspired by the remarkably hierarchical structure of sensory processing streams in cortex, where the laminar patterns of cortical-cortical connections define their function as ascending (forward or bottom-up) or descending (backward or top-down) connections and establish hierarchical relations between cortical areas (Felleman and Van Essen, 1991; Hilgetag et al., 2000). Computationally, the key idea of predictive coding is that cortical areas communicate in loops: Each area sends predictions about the activity in the next lower level of the hierarchy via backward connections; conversely, the lower level computes the difference or mismatch between this prediction and its actual activity and transmits the ensuing prediction error by forward connections to the higher level, where this error signal is used to update the prediction (Figures 2, 3). This recurrent message passing takes place across all levels of the hierarchy until prediction errors are minimized throughout the network. In the words of Rao and Ballard (1999): “[…] neural networks learn the statistical regularities of the natural world, signaling deviations from such regularities to higher processing centers. This reduces redundancy by removing the predictable, and hence redundant, components of the input signal.” This is a computationally attractive proposition because it satisfies information-theoretical criteria for a sparse code (Rao and Ballard, 1999).

In this scheme, minimizing prediction errors under the predictions encoded by the synaptic weights of backward connections in the hierarchy corresponds to hierarchical Bayesian inference and allows for computing the posterior probability of the causes, given the sensory data. Notably, plausible neuronal implementations exist which are compatible with known neuroanatomy and neurophysiology (Friston, 2005, 2008; Bastos et al., 2012); for a beautiful tutorial introduction (see Bogacz, in press).

A notion closely related to predictive coding is the idea that layers of hierarchical generative models may not predict the state of the next lower level, but its temporal evolution. This is known as hierarchical filtering and emphasizes the importance of taking into account the volatility of the environment, i.e., the temporal instability of its statistical structure, such as the probabilities by which one event causes another (Behrens et al., 2007; Mathys et al., 2011). Here, a hierarchical generative model combines a lower layer with value prediction errors about environmental variables with upper layers where volatility prediction errors drive inference and learning (Mathys et al., 2014). One concrete implementation of this idea is the hierarchical Gaussian filter (HGF; Mathys et al., 2011, 2014) which allows one to estimate subject-specific parameters encoding an individual's approximation to Bayes-optimal hierarchical learning.

One property of hierarchical Bayesian models deserves particular emphasis. This is the fact that under broad assumptions (i.e., for all distributions from the exponential family; Mathys, 2016), hierarchical Bayesian belief updates have a generic form with remarkably simple interpretability: at any given level *i*, belief updates Δμ_*i*_ are proportional to the prediction error (sent from the level below) but weighted by uncertainty or, more specifically, a precision ratio (Figure 3). This ratio denotes the relation between the estimated precision of the input from the level below (e.g., signal-to-noise ratio of a sensory input) and the precision of the prior belief. For example, in the case of the HGF, this takes the following form:

(3) $$\begin{array}{r} \Delta \mu_{i}\;\propto \;\frac{{\hat{\pi }}_{i-1}}{\pi_{i}}\;PE_{i-1} \end{array}$$

Here, the numerator of this precision ratio represents the expected precision of the input from the level below (i.e., the agent's estimate of signal-to noise ratio of the input), whereas the denominator encodes the precision of the current belief. That is, the impact of prediction error on a belief update is smaller the more precise (less uncertain) the prior belief and larger the more precise (higher signal-to-noise) the input from the level below. Evidence from anatomical and physiological studies has established bridges between the computational and physiological components of this hierarchical precision-weighted message passing: prediction error signaling via forward connections likely rests on glutamatergic (AMPA and NMDA) receptors, prediction signaling via backward connections probably exclusively on NMDA receptors, while precision-weighting is assumed to draw on mechanisms which modulate postsynaptic gain, such as neuromodulatory (e.g., dopamine or acetylcholine) or local GABAergic inputs (Friston, 2009; Corlett et al., 2010; Adams et al., 2013b); for a summary, see Figure 3.

### Perceptual control theory and active inference

Predictive coding represents one particular instantiation of the Bayesian brain hypothesis that represents an attractive foundation for studying interoception (Seth, 2013). However, predictive coding is limited to perception and does not directly speak to action selection and control, which is of fundamental importance for homeostasis. However, the link between perception and action can be studied in the framework of related theories which share the core ideas of predictive coding but generalize it to action selection; these include perceptual control theory (PCT; Powers, 1973, 1978; and active inference Friston, 2009; Friston K. J. et al., 2010).

**Figure 4:**
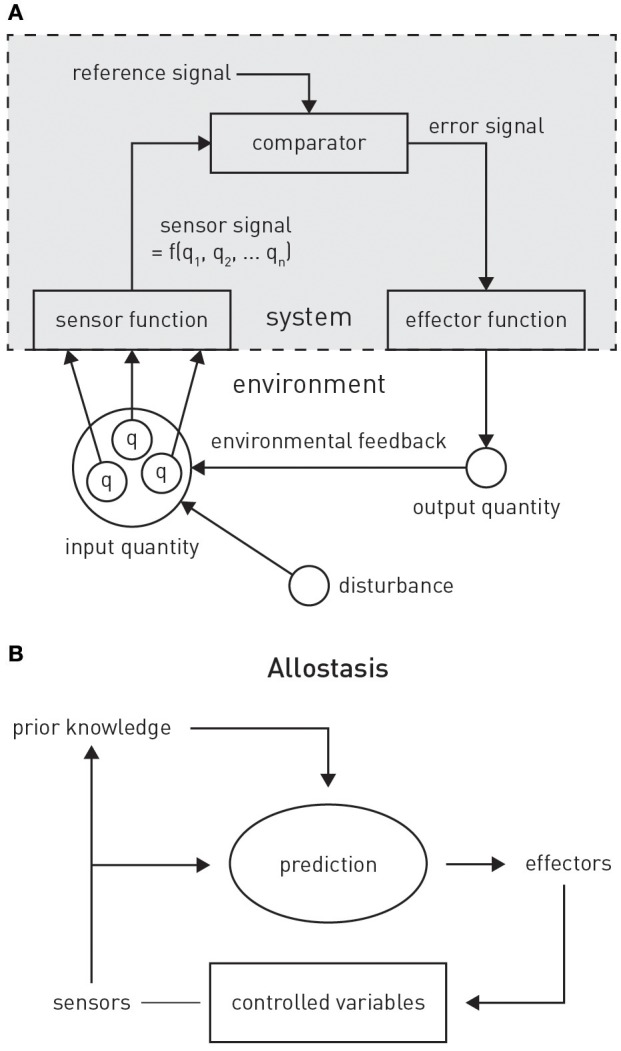
**(A)** Principles of classical feedback control. Figure is reproduced, with permission, from Powers (1973). **(B)** A graphical summary of allostasis and its dependence on predictions about future bodily states. Figure is reproduced, with permission, from Sterling (2012).

PCT originated from the control theoretic principles of cybernetics (Wiener, 1948) and cognitive theories emphasizing the self-referential structure of the brain, such as radical constructivism (Richards and von Glasersfeld, 1979; von Foerster, 2003). The central premise is that any adaptive system tries to control certain quantities in the environment, *q*, that are essential for the system's existence and survival (Figure 4A). Critically, as it can only infer the value of *q* through perception, controlling *q* amounts to ensuring that the sensory inputs reflecting *q* remain at the desired (expected) level. In other words, the system will resist any external perturbations or disturbances by eliciting appropriate actions that restore the expected sensory input. This control can be exerted by the classical negative feedback loop of cybernetics (Figure 4A), where an internal reference (setpoint or goal signal) is compared to incoming sensory input reflecting the state of *q*. The resulting mismatch or prediction error serves to elicit actions which restore *q* to the expected value. In Powers' (1973) words: “The reference signal is a *model* [our emphasis] inside the behaving system against which the sensor signal is compared: behavior is always such as to keep the sensor signal close to the setting of this reference signal.”

Critically, PCT postulates that control systems are, in many cases, structured hierarchically, where the “action” of higher systems consists of providing the reference or goal signal for lower systems. As a consequence, in order to reach a high-order goal, the relevant systems level (say *i*) does not need to directly access any actuators or specify a chain of commands; all it has to do is to alter the reference signal for the next lower system *i* −1. This will adjust the output from *i* −1 and thus the reference signal for the next lower system *i* −2, and so forth, until a level is reached whose output drives actuators and thus impacts on the environment. Intriguingly, nowhere in this chain of downward changes is the actual behavioral act specified; it is only the goals (expected sensory inputs) that are re-specified at each level of the hierarchy when the sensed environmental state does not correspond to the goal state (reference signal) at any levels of the hierarchy. In a nutshell, “…control systems control what they *sense*, not what they *do*.” (Powers, 1973; his emphasis).

PCT was formulated at a time when neither the hierarchical structure of the human brain was well understood, nor when Bayesian ideas of perception had been well developed. These concepts have informed a more general framework—active inference (Friston, 2009; Friston K. J. et al., 2010)—which, although not directly building on PCT, shares its fundamental notion that control is hierarchically organized and directed toward sensory input, not motor output. Active inference derives from the free energy principle (Friston et al., 2006; Friston, 2009, 2010) which postulates that biological agents strive to minimize surprise about their sensory inputs. In the general case, however, this requires integrating over all possible hidden states of the world, a computationally intractable problem. A solution is provided by a more easily computable quantity called “free energy” which represents an upper bound on surprise. A free energy minimizing system thus corresponds to a system which experiences minimum surprise about its sensory inputs. This notion is similar to the functional principle underlying PCT, but is formulated in terms of probability theory and thus tied closely to inference and generative models. The free energy principle has found numerous applications to cognition, suggesting efficient algorithms for how perceptual inference and learning can be implemented by a hierarchical generative model that maps onto the known neuroanatomy and neurophysiology of the cortex (Friston et al., 2006; Friston, 2008, 2009; Bogacz, in press).

Notably, the brain could reduce free energy in two major ways (Friston, 2009): (i) by updating its beliefs or expectations; this corresponds to adjusting its generative model of sensory inputs, as postulated by predictive coding; or (ii) by selecting those actions which lead to sensory inputs that are in accordance with the brain's expectations; this is active inference. Simply speaking, active inference suggests that predictions (prior expectations) about sensory inputs define preferences or goals that engender behavior (Friston et al., 2015). Similar to PCT, prior expectations at a high-level in the hierarchy define a set point against which current sensory input is evaluated and actions are automatically elicited by lower levels initiated until any mismatch is eliminated. That is, actions arise from a hierarchical cascade of changes in expectations that eventually lead to reflex-like motor behavior at the lowest level in order to yield the expected sensory input (Adams et al., 2012, 2013a; Friston et al., 2015).

## A hierarchical Bayesian view on fatigue and depression as meta-cognitive phenomena

### Circuit models of interoception and homeostatic control

Hierarchical Bayesian theories have begun to play an influential role in the treatment of interoception and homeostatic control. Although not specifying a particular computational mechanism, a seminal paper by Paulus and Stein (2006) highlighted the importance of predictive processes for understanding interoception and its role in psychopathology, specifically anxiety. More recently, several proposals have linked interoception and homeostatic/allostatic control to predictive coding and active inference (Seth et al., 2011; Gu et al., 2013; Seth, 2013; Feldman-Barrett and Simmons, 2015).

While these proposals have remained unspecific about the exact implementation of active inference for allostatic control, they have incorporated anatomical and physiological knowledge about the neuronal circuits for interoception and homeostatic control (for reviews, see Saper, 2002; Craig, 2002, 2003; Critchley and Harrison, 2013). Viscerosensory information about a wide range of bodily states—including bodily integrity (pain, inflammatory mediators), cardiovascular (e.g., blood pressure, oxygenation), humoral (e.g., plasma osmolality), physical (e.g., body temperature), metabolic (e.g., levels of glucose and hormones like insulin, ghrelin, leptin), immunological (e.g., cytokines), or mechanical (e.g., dilation of internal organs) properties—reaches the brain via three main channels: visceral afferents that enter the spino-thalamic tract via spinal cord lamina 1, cranial nerves IX (glossopharyngeal) and X (vagus), and humoral information which is sensed by circumventricular organs and specialized hypothalamic neurons situated outside the blood-brain barrier. These channels reach the thalamus (ventroposterior and ventromedial nuclei)—either directly or indirectly via brain stem nuclei including the nucleus of the solitary tract, parabrachial nucleus, and periaqueductal gray—and eventually target the viscerosensory cortex. The latter essentially comprises posterior and mid-insular cortex which represent a viscerotopic map of bodily state with respect to numerous physiological variables (Cechetto and Saper, 1987; Allen et al., 1991; Craig, 2002). Their efferent connections convey information about bodily state to cortical visceromotor areas—such as anterior insular cortex (AIC), anterior cingulate cortex (ACC), subgenual cortex (SGC), and orbitofrontal cortex (OFC)—which, in turn, send projections to hypothalamus, brainstem and spinal cord nuclei (Mesulam and Mufson, 1982; Hurley et al., 1991; Carmichael and Price, 1995; Freedman et al., 2000; Chiba et al., 2001; Vogt, 2005; Hsu and Price, 2007) in order to control autonomic, endocrine and immunological reflex arcs.

Based on this general anatomical layout, several computationally inspired proposals have been put forward, although so far without mathematically concrete implementations. Seth et al. (2011) conceptualized interoception as a predictive coding process combined with corollary discharge. In their concept, the AIC was assigned a central role as comparator (Gray et al., 2007) receiving corollary discharges (efference copies) of autonomic control signals from visceromotor regions like the ACC. Subsequent formulations based on active inference no longer understood autonomic control signals originating from visceromotor regions as “commands,” but as predictions of bodily states which are fulfilled by autonomic reflexes implemented by lower (hypothalamic and brainstem) centers (Gu et al., 2013; Seth, 2013; Feldman-Barrett and Simmons, 2015; Pezzulo et al., 2015).

In the following, we build on and extend the above models to formulate a theory of fatigue that connects hierarchical Bayesian inference to metacognition (cognition about cognition). Specifically, we unpack and extend a mechanism proposed recently as part of a list of priority problems for psychiatry: “With respect to fatigue, can we identify distinct patient subgroups in whom the brain's model of interoceptive inputs signal constant surprise because of persistent violation of fixed beliefs (homoeostatic setpoints) regarding metabolic states or bodily integrity and in whom this enduring dyshomoeostasis induces high-order beliefs about lack of control and low self-efficacy?” (Problem 8 in Stephan et al., 2016a). In the following, we describe how homeostatic regulation can be regarded as a problem of hierarchical Bayesian inference and control, not dissimilar to previous accounts but with three novel aspects: (i) an explicit discussion of how conventional homeostatic concepts can be transformed into Bayesian counterparts, including an extremely simple but concrete illustration of how active inference could mediate homeostatic control; (ii) the extension of active inference to a formal definition of allostatic control; and (iii) the addition of a metacognitive layer to the interoceptive hierarchy.

### Homeostatic control through active inference as “Bayesian reflexes”

The conventional cybernetic view of homeostasis regards the brain's task as ensuring that its carrier (the body) inhabits a limited number of states which are compatible with survival, for example, being within a narrow range of body temperature or blood oxygenation. From a control theory perspective, this amounts to keeping sensory signals of bodily state close to setpoints, which are defined by a reference signal that is provided to a comparator unit (compare the traditional cybernetic circuit implementing feedback control in Figure 4A).

We now formulate this circuit and its components in a way that provides a basis for extending homeostatic to allostatic control. Under a Bayesian perspective, homeostatic setpoints can be defined as the expectations (means) of prior beliefs about the states the body should inhabit. These prior beliefs are instantiated biophysically by “hard-wired” local circuits in “effector regions” that control homeostatic reflex arcs, such as the hypothalamus, brain stem nuclei like the periaqueductal gray (PAG), and autonomic cell columns in the spinal cord (Craig, 2003). The “homeostatic range” (of values compatible with life) are reflected by prior variance: priors of vitally important variables (such as blood oxygenation) are extremely tight (low variance), whereas other beliefs (e.g., about blood pressure) can afford being considerably wider (high prior variance). Notably, all of these beliefs are subject to evolutionary pressure: depending on how well they support homeostasis under the conditions of a given environment and thus maintain bodily integrity and survival, they (the neuronal structures encoding them, respectively) will be more or less likely selected out.

**Figure 5:**
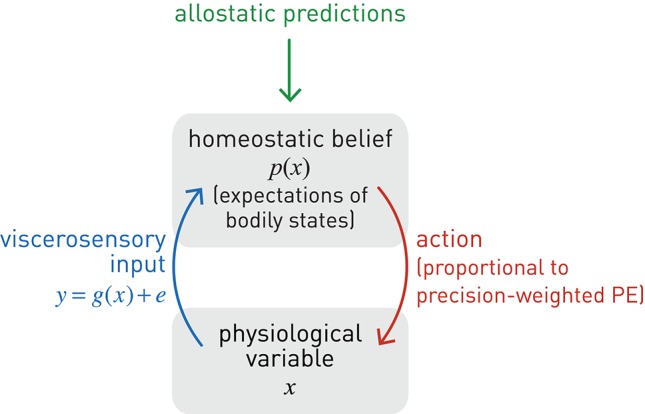
**A graphical summary of a homeostatic reflex arc and its modulation by allostatic predictions**. Blue lines: sensory inputs; red lines: prediction errors; green lines: predictions.

Given this notion of a homeostatic setpoint as a belief about physiological states the body should inhabit, one can now formulate a basic homeostatic reflex arc in Bayesian terms (Figure 5), including its control by higher-order centers (allostasis). Specifically, we consider how a particular physiological state *x* can be controlled by actions elicited by a neuronal homeostatic reflex arc, e.g., in the hypothalamus or a brain stem nucleus, ensuring that *x* is kept within a homeostatic range (prior belief about its value). A key property of this formulation is that corrective action is more vigorous or rapid the higher the deviation of bodily states from prior expectations *and* the more precise these expectations (the tighter the homeostatic range). For clarity and simplicity, we only consider a very basic scenario here. While our approach is inspired by more general and sophisticated treatments of active inference formulated under the free-energy principle (Friston K. J. et al., 2010), the following derivation presents, to our knowledge, a first mathematically concrete proposal of an active inference mechanism for homeostatic reflexes under allostatic control. We emphasize that the following model is by no means complete, but should be seen as a mere starting point for developing generative models of allostatic control and metacognitive evaluation.

Let us initially begin from the perspective of perceptual inference as Bayesian belief updating, i.e., how one could determine the most likely value of bodily state *x*, given noisy sensory input which is sampled sequentially. Here, we examine the simplest case where *x* is assumed not to evolve or experience any perturbations over the period of observation. While, in this context, *x* is thus a constant state of the body, the brain's belief about *x* is updated sequentially, based on noisy sensory inputs (Because of this subtle difference, in the following few paragraphs on perception, we write the bodily state x as a time-invariant variable while mean and variance of the belief about x are time-dependent. In subsequent paragraphs on action, the opposite is the case). This belief can be described, for example, as a normal distribution with mean μ_*t*_ and precision (inverse variance) π_*t*_ at time *t*:

(4) $$\begin{array}{r} p\left(x\right)=N\left(x;\mu_{t},\pi_{t}^{-1}\right) \end{array}$$

At any time *t*, viscerosensory input *y* results from some form of neuronal coding (transformation) *g* of *x* and is affected by inherent noise of the sensory channel (with constant precision π_*data*_):

(5) $$\begin{array}{r} p\left(y|x\right)=N\left(y;g\left(x\right),\pi_{data}^{-1}\right) \end{array}$$

or, equivalently:

(6) $$\begin{array}{r} y_{t}\;=\;g\left(x\right)+e_{t} \end{array}$$

$$\begin{array}{r} p\left(e_{t}\right)\;=\;N\left(e_{t};0,\pi_{data}^{-1}\right) \end{array}$$

In this context, the goal of perceptual inference would be to infer on the value of *x* given repeated samples of the noisy viscerosensory signal *y*. This corresponds to updating ones' estimates of the sufficient statistics of *x* (μ_*t*_ and π_*t*_), where the estimate at time *t* serves as the prior for the next belief update (“today's posterior is tomorrow's prior”). That is, using Bayes' theorem, one can sequentially transform a prior belief $p\left(x;\mu_{t},\pi_{t}^{-1}\right)$ into a posterior belief $p\left(x;\mu_{t+1},\pi_{t+1}^{-1}\right)$, based on new sensory data *y*_*t*_. Specifically, this sequential belief update would obey the following simple rule (see Mathys, 2016 for details):

(7) $$\begin{array}{l} \mu_{t+1}=\mu_{t}+\frac{\pi_{data}}{\pi_{t}+\pi_{data}}\left(y_{t}-g\left(\mu_{t}\right)\right) \end{array}$$

$$\begin{array}{l} \pi_{t+1}=\pi_{t}+\pi_{data} \end{array}$$

Here, the posterior mean results from updating the current estimate (prediction or prior mean) with the precision-weighted prediction error—where the latter corresponds to the difference between the actual sensory signal *y*_*t*_ and its predicted value, *g*(μ_*t*_). The precision-weighting is critical because it renders the correction or update sensitive to the properties of both the sensory channel and the prior belief: the belief update is more pronounced the higher the estimated precision of the sensory input and the lower the precision of the prior.

We can now use the same type of precision-weighted prediction error for influencing *x*, instead of inferring or sensing it. In other words, we turn the perceptual update rule of Equation 7 into a control rule, based on two simple considerations. First, to fix the setpoint for the homeostatic reflex, we clamp the prior belief: ∀*t*:μ_*t*_ = μ_*prior*_, π_*t*_ = π_*prior*_. This effectively corresponds to delta function (hyper)priors on the sufficient statistics of the prior belief (see Equation 8). Equation (7) shows that this can be achieved by simply ignoring the sensory information (more formally: setting the data precision to zero). Second, we define an action or effector function whose driving force is the prediction error under expected homeostasis; in other words, the difference between the actual sensory input *y* and the sensory input that would be expected at the homeostatic setpoint (μ_*prior*_). This prediction error can be derived from the log evidence of a model *m*_*H*_ which expects bodily state to be in homeostasis [and therefore the viscerosensory input to equal *g*(μ_*prior*_)]. This is the case when the sufficient statistics of the marginal likelihood are given by the homeostatic setpoint μ_*prior*_ and homeostatic range $\pi_{prior}^{-1}$ (where *c* absorbs constant terms):

(8) $$\begin{array}{r} p\left(y|m_{H}\right)=\int p\left(y|\mu_{t},\pi_{t}\right)p\left(\mu_{t}\right)p\left(\pi_{t}\right)d\mu_{t}d\pi_{t}\\ =\int N\left(y;g\left(\mu_{t}\right),\pi_{t}^{-1}\right)\delta \left(\mu_{t}-\mu_{prior}\right)\\ \delta \left(\pi_{t}-\pi_{prior}\right)d\mu_{t}d\pi_{t}\\ =N\left(y;g\left(\mu_{prior}\right),\pi_{prior}^{-1}\right)\\ L=\ln\;p\left(y|m_{H}\right) \end{array}$$

$$\begin{array}{r} =\frac{1}{2}\left(\ln\pi_{prior}-\pi_{prior}{\left(y-g\left(\mu_{prior}\right)\right)}^{2}\right)+c\\ =\frac{1}{2}\left(\ln\pi_{prior}-\pi_{prior}{\left(PE\left(y\right)\right)}^{2}\right)+c \end{array}$$

Notably, this is the negative (Shannon) surprise *S* of seeing the data under the expectation of homeostasis (compare Equation 2):

(9) $$\begin{array}{r} S\left(y|m_{H}\right)=-L \end{array}$$

According to Equation (8), minimizing the precision-weighted squared prediction error thus minimizes the interoceptive surprise *S* about the sensory inputs. This requires actions that make *x* maximally congruent with the homeostatic setpoint and hence maximize log evidence *L*. This can be achieved by defining action^4^ as the gradient of the log likelihood with regard to *x* (under application of the chain rule and noting, from Equation (6), that $\frac{\partial y}{\partial x}=\frac{\partial g}{\partial x}$):

(10) $$\begin{array}{l} a\left(t\right)=\frac{\partial L}{\partial x}\;\\ =\frac{\partial \left[-\frac{1}{2}\pi_{prior}{\left(y\left(x,t\right)-g\left(\mu_{prior}\right)\right)}^{2}\right]}{\partial x}\;\\ =\frac{\partial \left[-\frac{1}{2}\pi_{prior}PE{\left(y\right)}^{2}\right]}{\partial x}\;\\ =-\frac{\pi_{prior}}{2}\frac{\partial \left[PE{\left(y\right)}^{2}\right]}{\partial y}\frac{\partial y}{\partial x} \end{array}$$

$$\begin{array}{l} =-\pi_{prior}PE\left(y\right)\frac{\partial g}{\partial x} \end{array}$$

and using it to smoothly adjust the value of the physiological variable *x*:

(11) $$\begin{array}{r} \frac{\text{d}x}{\text{d}t}=\lambda^{-1}f\left(a\left(t\right)\right) \end{array}$$

Put differently, the chosen action *a* induces a gradient descent of *x* on interoceptive surprise:

(12) $$\begin{array}{r} \frac{\text{d}x}{\text{d}t}=-\lambda^{-1}f\left(\frac{\partial S}{\partial x}\right) \end{array}$$

**Figure 6:**
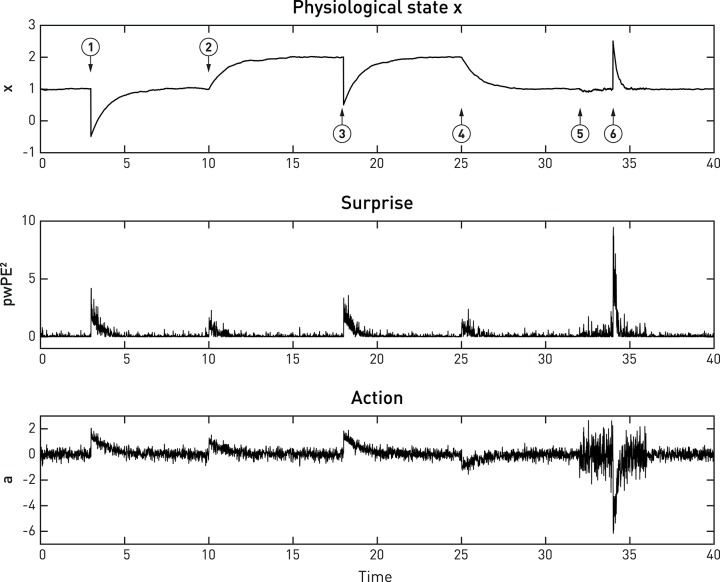
**A simulated example of allostatic regulation of homeostatic control, based on Equations (8)–(12)**. The upper panel shows the temporal evolution of a fictitious physiological state *x* (Equation 11) which is affected by environmental perturbations (➊,➌,➏; all with a magnitude of 1.5). The middle and lower panels display an approximation to interoceptive surprise—i.e., squared precision-weighted prediction error (pwPE^2^; compare last line of Equation 8)—and the associated action signal (Equation 10), respectively. Following the timeline from left to right, the homeostatic setpoint or belief is initially specified with a prior mean and prior precision of 1 each. Please note that even before the first perturbation (➊) occurs, sensory noise (zero mean, 0.25 standard deviation) leads to ongoing actions of minute amplitude which lead to (very small) deviations of *x* from the setpoint. Following a first perturbation (➊), the homeostatic reflex arc emits corrective actions that are proportional to precision-weighted viscerosensory prediction error (middle panel). As the actions are successful, *x* returns to setpoint and viscerosensory prediction error decays. ➋ indicates the beginning of allostatic control: here, the prediction of imminent future perturbations (by some generative model not specified here) leads to an anticipatory rise in the homeostatic setpoint (a shift in the prior mean to 2). As a consequence, in the absence of any change in sensory input, actions are elicited to change the value of *x* to the new setpoint. This ensures that the following perturbation ➌ does not bring *x* anywhere near the critical threshold. At ➍, a safe period is predicted, and allostatic control resets the homeostatic setpoint (prior mean) to 1. At ➎, another perturbation in the near future is being predicted, however, this time the direction of the perturbation is uncertain. Therefore, changing the mean or setpoint is not a viable option and allostatic control takes a different form: instead of changing prior mean, the prior precision of the homeostatic belief is increased from 1 to 4. As a consequence, when a perturbation occurs at ➏, this yields a considerably larger precision-weighted prediction error and hence greater interoceptive surprise (see lower panel), leading to a significantly more rapid corrective action (compare the slope of signal rise between ➊ and ➏), putting the agent at less risk, should another perturbation occur shortly after ➏. It is also noteworthy that the increased prior precision enhances the effect of sensory noise (compare the roughness of the three signals just prior to ➊ and ➏, respectively).

Here, λ is a time constant matched to the time scale at which action can affect *x* (for example, a slow time constant for hormonal regulation by the hypothalamus, or a very fast time constant for cardiovascular regulation via the baroreceptor reflex). Furthermore, for generality, Equation (11) includes a mapping *f* from action to changes in *x*. This could be non-linear and probabilistic to account for noise in motor processes (compare the analogous sensory mapping *g* in Equation 6). The advantage of a probabilistic formulation is that it allows for considering “action precision,” i.e., the confidence with which an action would have the desired effect on the physiological variable; this will be examined in future work. In the present simulation shown in Figure 6, we have kept *f* maximally simple (a deterministic identity function).

Equations (8)–(12) specify how the effector emits actions that move *x* toward its setpoint and minimize precision-weighted prediction error and thus interoceptive surprise (see middle and lower panels in Figure 6). This makes the action signal progressively diminish toward zero as *x* asymptotes its setpoint. Notably, the vigor or speed of action depends on both the current prediction error (discrepancy between the sensory feedback signal and its desired/predicted level), and the precision of the prior homeostatic belief. This means that for vitally important physiological variables whose homeostatic ranges are very tight, corrective actions are necessarily rapid. Conversely, when physiological variables diverge from setpoints, the experience of dyshomeostasis (i.e., the magnitude of prediction error) is much more pronounced when prior homeostatic beliefs are tight. Both properties are illustrated by the simple simulation shown in Figure 6.

The above equations illustrate a key principle of active inference: the choice between reducing prediction error through changing predictions (updates of the generative model) or through action depends on precision. For example, reducing the precision of sensory input (π_*data*_ in Equation 7) disables belief updates while action (Equation 10) remains unaffected. Similarly, increasing the precision of predictions or prior beliefs (π_*t*_ in Equation 7 or π_*prior*_ in Equation 8) abolishes belief updating while action is increased in proportion to the increase in precision. In other words, a modulation of precision of top-down predictions is sufficient to switch from learning to acting.

This section has outlined a Bayesian account of homeostatic control. Equations (8)–(12) illustrate the role of prior beliefs for implementing setpoints in homeostatic reflex arcs where actions minimize prediction errors (and hence interoceptive surprise) in order to fulfill a prior belief that physiological state *x* should be within a particular range. This represents a simple but concrete implementation of active inference in the context of homeostatic control. Perhaps most importantly, dyshomeostasis can now be defined formally as a persistent deviation from precise prior expectations about bodily state that is indexed by chronically elevated surprise about viscerosensory inputs; or equivalently, as high entropy (average surprise) of viscerosensory channels.

One might note that entropy minimization by homeostatic control might constitute a violation of the second law of thermodynamics (that all systems monotonically increase their entropy over time). However, the second law of thermodynamics only applies to closed systems; by contrast, biological organisms represent open systems which exchange energy and information with their environment and are capable of decreasing entropy—at least temporarily (Von Bertalanffy, 1969). This is the very nature of homeostatic regulation: to maintain the body in a highly particular (low entropy and hence unlikely) condition.

### An active inference perspective on allostasis

A critical extension of the above scheme for homeostatic control is to allow higher-order goals or predictions to alter the homeostatic belief *p*(*x*). This amounts to allostasis: the proactive deployment of behavior, guided by predictions from a model, in order to avoid dyshomeostatic future states (Sterling, 2012; Figure 4B). For example, prolonged exposure to intense sunlight will not only cause immediate (e.g., increase in body temperature) but also delayed (e.g., dehydration) perturbations of homeostasis. Provided the brain is equipped with a generative model for predicting the evolution of environmental and bodily states, based on previous experience, it can take proactive actions and avoid dyshomeostatic states before they arise. Importantly, these homeostatic goals often have a hierarchical structure, where temporary deviations from homeostatic setpoints are tolerated or even induced in order to ensure that higher-order homeostatic goals can be reached in the future. For example, under a model that predicts a possible encounter with a hostile agent in a specific context, anticipatory deviations from hormonal and cardiovascular setpoints are induced to prepare for future fight-flight behavior.

From the perspective of theories like PCT or active inference, an efficient way of accomplishing hierarchical control is to temporarily alter the setpoint or prior belief of the relevant homeostatic reflex arc (for example, changing the belief about desirable plasma osmolality elicits drinking behavior before dehydration reaches a critical level). This relies on higher brain structures with three properties: (i) access to estimates of bodily state (interoception), (ii) capable of generating predictions over longer time scales, and (iii) with anatomical connections that can modulate the homeostatic beliefs which reflex arcs in regions like the hypothalamus or brain stem serve to fulfill. Neuroanatomically, regions that are in a position to modulate homeostatic reflex arcs through allostatic predictions are likely situated at the top of the interoceptive hierarchy and include the AIC, ACC and subgenual cortex; this is discussed in more detail below.

The hierarchical (top-down) modulation of reflex arcs by predictions means that (homeostatic) beliefs about desirable bodily states in the present become dependent on (allostatic) beliefs about bodily states in the future. This essentially turns homeostatic beliefs into time-varying quantities under the influence of higher allostatic predictions ϕ_*i*_(*t*). This belief transformation could affect either the mean and/or the precision of the homeostatic belief across time:

(13) $$p\left(x\left(t\right)\right)=N\left(x\left(t\right);\mu_{prior}\left(\phi_{1}\left(t\right)\right),\pi_{prior}{\left(\phi_{2}\left(t\right)\right)}^{-1}\right)$$

Figure 6 shows a very simple simulation which illustrates both types of allostatic control. Here, a physiological variable *x* is driven away from its setpoint (the agent's homeostatic belief) three times, due to environmental perturbations. Each time a “Bayesian reflex” restores homeostasis according to Equations (8)–(12). Critically, following the first incident (➊ in Figure 6), higher levels of the system (not modeled here) predict further perturbations of a particular direction and allostatic control is exerted by shifting the mean (setpoint) into the opposite direction while leaving the precision of the homeostatic belief unaffected (➋). As a consequence, *x* rises to the new expected level. Note that this occurs without specifying the action; instead, the action follows automatically once a new belief or setpoint has been adopted. At ➎, perturbations are predicted, but with uncertainty about their direction; hence shifting the setpoint or mean is not a viable option. Instead, the precision of the homeostatic belief is increased, leading to a smaller range of tolerated deviations in either direction. The subsequent response to a perturbation (➏) leads to a far swifter restorative response than after the first perturbation (➊).

Here, we only provide a general frame for implementing allostasis from an active inference perspective; the specific form for the modulation of homeostatic beliefs by allostatic predictions is likely to vary across physiological variables, as these are controlled on different time scales and may draw on predictions from different generative models. Generally, however, we note that the frame suggested by Equation (13) is consistent with the PCT notion of control where hierarchically higher levels set the reference points for lower levels (Powers, 1973). It is also similar in structure to active inference accounts of motor control where primary motor cortex is assumed to modulate spinal reflex arcs through ascending connections to α and γ motor neurons, “programming” motor trajectories via predictions about future proprioceptive input (Adams et al., 2013b).

In summary, under the hierarchical Bayesian view presented above, homeostatic and allostatic control, respectively, can be understood as active inference about bodily states on different time scales: actions (of a motor, autonomic, endocrine, or immunological sort) are selected which fulfill beliefs about current and future bodily states and reduce the average surprise (entropy) of viscerosensory channels over time (the time scale of the respective allostatic goal). Notably, this entropy-reducing principle may not only be in operation during the lifetime of an organism, but has also been suggested as the driving force behind the evolution of homeostatic mechanisms (Woods and Wilson, 2013).

### A neuroanatomical circuit for interoception, homeostasis, and allostasis

**Figure 7:**
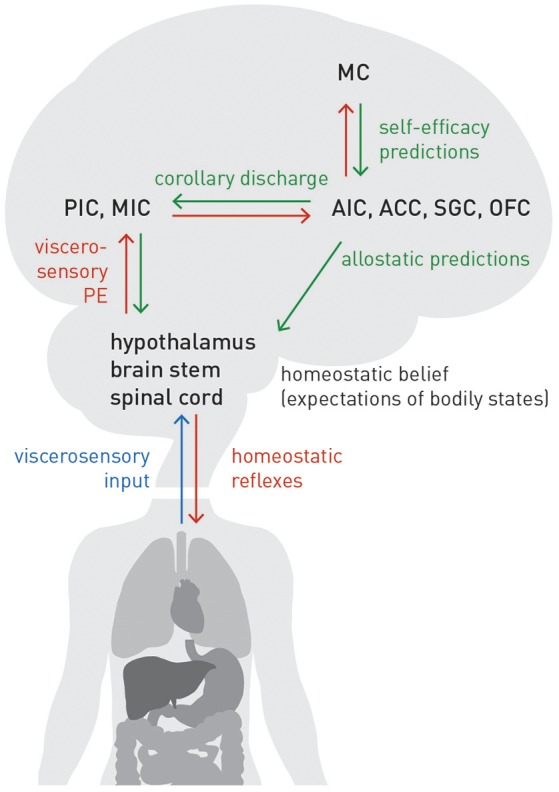
**A proposed circuit for interoception and allostatic regulation of homeostatic reflex arcs, together with a metacognitive layer (MC)**. See main text for details. Blue lines: sensory inputs; red lines: prediction errors; green lines: predictions.

The extension from homeostatic to allostatic control highlights that interoception and homeostatic regulation are inevitably linked and form a closed loop: tuning the setpoints of homeostatic reflex arcs depends on accurate allostatic predictions about future bodily states; these predictions, in, turn depend on accurate inference about current bodily states. Figure 7 summarizes the neuroanatomy of a proposed circuit for integrating the afferent (interoceptive) and efferent (control) branches of homeostatic/allostatic regulation. This anatomical layout is not dissimilar to a previous proposal by Feldman-Barrett and Simmons (2015) but introduces several novel aspects (e.g., a metacognitive layer) and distinguishes interoception, allostatic predictions and homeostatic reflex arcs more explicitly.

In our proposal, AIC, ACC, subgenual cortex (SGC), and orbitofrontal cortex (OFC)—regions we refer to as “visceromotor areas” (VMAs) as a set—are situated at the top of this circuit, embodying a generative model of (potentially different types of) viscerosensory inputs that enables a biological agent to infer on current bodily states and predict future states, as a basis for allostatic predictions. This assumption is supported by known anatomical connections and their hierarchical relations based on laminar patterns of origin and target: tract tracing studies in the Macaque monkey (Mesulam and Mufson, 1982; Mufson and Mesulam, 1982; Vogt and Pandya, 1987; Carmichael and Price, 1995) demonstrated that VMAs receive ascending projections from viscerosensory cortex (posterior and mid-insula). As in circuits supporting exteroception, these ascending connections are thought to signal prediction errors (Seth et al., 2011; Gu et al., 2013; Seth, 2013; Feldman-Barrett and Simmons, 2015). On the other hand, according to tract tracing studies in monkeys and rats (Mesulam and Mufson, 1982; Hurley et al., 1991; Carmichael and Price, 1995; Freedman et al., 2000; Chiba et al., 2001; Vogt, 2005; Hsu and Price, 2007), the visceromotor areas possess connections targeting hypothalamus, brain stem nuclei and spinal cord (partially relayed by amygdala, periaqueductal gray (PAG), and basal ganglia). These connections are thought to convey allostatic predictions which modulate the setpoints of homeostatic reflexes, as described above. Importantly, descending projections from visceromotor areas could send the same prediction to posterior and mid-insula; this effectively serves as efference copy or corollary discharge against which viscerosensory inputs can be compared. The resulting prediction errors are returned via ascending connections to visceromotor areas, allowing for (presumably slow) adjustment of allostatic predictions.

Several sources of uncertainty need to be highlighted here. First, the specific roles and division of labor amongst VMAs are largely unclear; for the moment, we have grouped them together without any differentiation. Second, non-trivial species differences in the neuroanatomy of interoceptive circuitry exist. For example, SGC targets different autonomic effector regions in rodents and monkeys (Hurley et al., 1991; Freedman et al., 2000), and it has been questioned whether AIC and ACC in monkeys and humans are functionally equivalent (Critchley and Harrison, 2013). Third, the present circuit model ignores the fact that AIC, ACC, and OFC each consist of several anatomically distinct subfields. For example, even within agranular insular cortex of the Macaque monkey, subareas exhibit differential connectivity and may possess a hierarchical relation amongst themselves (Carmichael and Price, 1996). Finally, our present model assumes that effector regions (hypothalamus, brain stem, spinal cord), which receive allostatic predictions from VMAs, do not return prediction errors via ascending connections. This serves to ensure that allostatic predictions are fulfilled by actions, instead of these predictions being revised by prediction errors. This fits well to the agranular cytoarchitectonic nature of VMAs, i.e., the absence of a well-formed layer IV which represents a key target lamina for ascending connections conveying prediction errors in granular cortex (cf. Feldman-Barrett and Simmons, 2015). Alternatively, as suggested by analogous active inference schemes for motor control, this type of prediction error signal could temporarily “switched off” or attenuated during action execution by reducing precision (Adams et al., 2012, 2013a). It is questionable, however, whether this proposed mechanism could also apply to allostatic control, given the continuous presence of interoactions and the much longer time scales on they unfold (e.g., hormonal or immunological regulation).

Our Bayesian account of homeostatic control (see Equations 8–12 above) led to a definition of dyshomeostasis as a state of elevated interoceptive surprise, a deviation from precise prior expectations about bodily state that is indexed by increased precision-weighted prediction errors about viscerosensory inputs. The closed perception-action loop of homeostatic inference and control shown by Figure 7 indicates that, in addition to peripheral causes residing in the body itself, structural lesions (e.g., due to demyelinating processes in MS) or functional impairments (e.g., due to inflammation in depression) in either afferent or efferent branches of this circuit could lead to chronic dyshomeostasis. We now turn to some implications of this view for specific domains of cognition and emotion: fatigue and depression.

### Metacognition about interoception and allostatic self-efficacy

Interoceptive surprise plausibly has general and major consequences for cognition and emotion—even in interoceptive domains that may be operating outside immediate awareness (e.g., levels of certain hormones, cytokines, or metabolites). As noted above, surprise is equivalent to negative log model evidence, and persistently high surprise is the hallmark of a bad model. A chronic state of perceived dyshomeostasis indicates that the brain's generative model of viscerosensory inputs has low evidence—either because it generates bad predictions or because it cannot transform them (with sufficient confidence) into homeostasis-restoring actions. In other words, persistently high interoceptive surprise represents a fundamental warning sign that the brain presently cannot adequately control perturbations of potential relevance for survival. This leads us to a key hypothesis of this paper—that “enduring dyshomoeostasis induces high-order beliefs about lack of control and low self-efficacy” (Stephan et al., 2016a).

Self-efficacy is a concept of self-evaluation and behavioral change which holds that humans not only have expectations with regard to the outcome of chosen actions, but also self-oriented expectations concerning whether they can successfully execute these actions (Bandura, 1977). Self-efficacy can be defined as an individual's expectation of personal mastery and control: an individual with high self-efficacy believes that he/she can successfully perform the cognitive and motor operations required to overcome negative situations (e.g., obstacles, adversaries, threats, and aversive experiences). The construct of self-efficacy is thus closely related to concepts of metacognition (for review, see Clark and Dumas, 2015). Theoretical and empirical work suggests that low levels of perceived self-efficacy prevent the deployment of adequate coping behavior and may constitute an important component in the pathogenesis of depression and anxiety (Rosenbaum and Hadari, 1985; Bandura et al., 1996, 1999; Arnstein et al., 1999).

While the importance of self-efficacy for adaptive behavior and general well-being has been examined in numerous cognitive domains, particularly with regard to learning, memory and other academically relevant cognitive skills, the possible link of self-efficacy to dyshomeostasis has received relatively little attention. One exception is the area of chronic pain research, where several studies demonstrated that perceived self-efficacy not only modulates pain perception (Bandura et al., 1987), but crucially determines coping behavior and quality of life, independently of and often more strongly than physical variables, such as pain intensity or duration (Arnstein et al., 1999; Denison et al., 2004; Burke et al., 2015).

Here, we suggest that the metacognitive evaluation of homeostatic/allostatic control during experienced dyshomoeostasis^5^ has a major impact on self-efficacy beliefs and the ensuing choice of actions. Importantly, as we highlighted at the outset of this paper, this may proceed in two sequential stages. Initially, the metacognitive recognition that available homeostatic/allostatic control strategies fail to reduce interoceptive prediction error may materialize through fatigue as a subjective feeling. This resonates with the concept of “feeling states” in the interoception literature (i.e., re-representations of an image of bodily state; Craig, 2002) but highlights the evaluation of action outcomes and the experience of mastery. Importantly, this can be defined formally under our model above: if the gradient $\frac{\partial S}{\partial x}$ (Equations 8–12) indicates that interoceptive surprise is not decreasing but maintains constant or even increases as the action is performed, this indicates that homeostatic control fails and the available action does not control the dyshomeostasis-causing process. Fatigue may thus be understood as the metacognitive detection of an ongoing but fruitless effort of regulating bodily states that may manifest neurophysiologically as a failure to reduce incoming prediction errors to VMAs (Figure 7).

In this context, it is worth pointing out that, under our model, fatigue can be formally distinguished from tiredness. In case of tiredness, for example, from prolonged physical activity, interoceptive surprise arises from the concentration of metabolites such as lactic acid shifted away from their setpoints. In this case, however, a simple action is available: physical rest. This allows muscle metabolism to restore biochemical balance, which turns the gradient $\frac{\partial S}{\partial x}$ negative (Equations 8–12) and signals restoration of homeostasis by the chosen behavior. By contrast, in fatigue, physical rest does not have the same positive effect. From the view of our theory, where fatigue represents a metacognitive belief that arises from chronic experience of lack of mastery over bodily states, it is easy to explain why rest is not beneficial: when interoceptive surprise fails to decrease in the absence of actions, mastery cannot be experienced and the associated metacognitive beliefs cannot be adjusted. This may be a reason why, in some patients, graded exercise therapy can be helpful (Larun et al., 2016), perhaps because it gradually allows patients to experience mastery and restore self-efficacy.

Having said this, while rest is not directly effective against fatigue, it may not be the worst behavioral option as it prevents inefficient actions that do not target the origin of interoceptive surprise and would only require energy (something that in itself induces a positive gradient $\frac{\partial S}{\partial x}$). Put differently, fatigue could initially be an adaptive and functionally meaningful feeling state: if presently no homeostasis-restoring action strategies are (perceived to be) available, or the means for implementing these strategies are lacking, it may be a rational choice to reduce activity and save energy. Three cases may be worth distinguishing here, depending on whether the cause of dyshomeostasis resides in the body, the physical environment, or the social environment, respectively. First, in case of a bodily origin of dyshomeostasis, fatigue-driven passivity allows for saving and reallocating energy. An example of fatigue as an early and meaningful response to dyshomeostasis is “sickness behavior” during acute infections (Dantzer and Kelley, 2007), which is characterized by fatigue, lethargy, and social withdrawal. Sickness behavior is commonly interpreted as an adaptive response which promotes the conservation and reallocation of energy to immunological defense processes (for review, see Shattuck and Muehlenbein, 2015). Second, given a perceived lack of mastery over causes of dyshomeostasis in the physical environment, it may be a better choice to let the environment evolve and change by itself; with some probability, this may lead to more favorable conditions under which existing action plans can be implemented. Third, if the cause of dyshomeostasis resides in the social environment but cannot be influenced by actions, a passive “wait and watch” strategy may offer opportunities for extending the brain's generative model—and thus scope of possible actions—by observational learning from other agents' behavior.

However, if none of the three positive effects described above materialize and the experience of dyshomeostasis becomes chronic, this may initiate a second phase characterized by a generalization of low self-efficacy beliefs—akin to learned helplessness (Abramson et al., 1978)—and the onset of depression. More specifically, an agent's experience of enduring dyshomeostasis signals a fundamental lack of mastery and control (over bodily states and thus survival) which may generalize, from the allostatic domain to other cognitive domains that are crucial for self-evaluation, planning and action selection. This draws on previous empirical findings that subjective beliefs of low self-efficacy can generalize beyond the specific situation (Bandura, 1977; Burke et al., 2015) and might result in a domain-unspecific vulnerability: the self-fulfilling expectation that one generally lacks control and cannot deploy adequate coping behavior in response to adverse events. In other words, an agent's chronic experience of dyshomeostasis may induce a generalized sense of hopelessness, which makes any actions appear futile and which triggers the onset of depression.

To prevent misunderstandings, we would like to emphasize three things. First, we neither postulate a deterministic relation between chronic dyshomeostasis and depression nor do we claim that its aetiological importance is restricted to depression. Instead, we regard a dyshomeostasis-induced sense of low self-efficacy as weakening resilience to stress in general and thus a risk factor for many forms of psychopathology. While perceived low self-efficacy likely represents an inevitable consequence of persistent dyshomeostasis, various protective factors may prevent its spread to other cognitive domains and block the generalization to hopelessness. For example, intellectual abilities or social support may maintain a sense of mastery that shields against an all-encompassing feeling of loss of control. Additionally, the experience of dyshomeostasis is usually restricted to certain bodily states but not others, leaving the possibility of experiencing preserved allostatic mastery in some domains. Second, we do not claim that a dyshomeostasis-induced sense of low self-efficacy represents a single cause for the entire depression spectrum. Instead, we propose that it may play a particularly important role in melancholia, a subtype of depression with pronounced somatic symptoms and endocrine disturbances (Parker and Paterson, 2014) that differs physiologically from other forms of depression with respect to functional connectivity of visceromotor areas, including SGC and ACC (Guo et al., 2016). Indeed, model-based indices of individual interoception and allostatic control may provide a foundation for differential diagnosis and prognosis, a theme we return to below. Third, in its present form, our theory is not designed to explain the full spectrum of interactions between fatigue and depression. Longitudinal studies have shown that the causal relation between fatigue and depression is unlikely to be unidirectional, but that both act as independent risk factors for each other (Skapinakis et al., 2004). Focusing on patients with bodily conditions that cause chronic dyshomeostasis, our theory only considers one of these directions—from dyshomeostasis to fatigue to depression. It suggests a possible mechanism (a “learned helplessness”-like generalization of perceived low self-efficacy) for the progression from fatigue to depression and, as described below, points to neurophysiological markers (in terms of effective connectivity) that may distinguish “pure fatigue” from the combined presence of fatigue and depression. By contrast, our theory is less specific in offering a mechanistic explanation for the opposite direction, i.e., how fatigue may result from depression. However, our framework would not be incompatible with the possibility that external triggers of depression might instantiate false beliefs about self-efficacy that, once fulfilled and entrenched, lead to fatigue. In other words, in this case, the generalization of low self-efficacy believes would proceed in the opposite direction as discussed above, from various cognitive domains to homeostatic/allostatic control.

Viewing fatigue and depression as sequential consequences of the subjective belief of low self-efficacy with regard to homeostatic/allostatic control frames them as metacognitive phenomena. This implies that the hierarchical circuit for interoceptive inference and homeostatic/allostatic regulation discussed above likely represents only the lower level of a more complex system which includes a higher metacognitive layer for monitoring the performance of homeostatic/allostatic control (Figure 7). What exactly, however, is being monitored, and what could be the anatomical basis of this metacognitive layer?

### Anatomical and computational aspects of metacognition

To our knowledge, there presently exist no specific computational models of a metacognitive system for interoception and homeostatis/allostasis. Here, we outline two ideas of how such a model could look like, without going into mathematical detail. One possibility, shown in Figure 7, is that the metacognitive level simply represents another layer on top of the hierarchical circuit for interoception and homeostatic/allostatic control and follows the same hierarchical Bayesian principles. Specifically, this metacognitive layer (metacognitive cortex “MC” in Figure 7) would encode high-order beliefs about allostatic mastery, for example, the belief that one is capable of responding adaptively to any perturbation one may possibly experience. These beliefs at the metacognitive level would then serve as predictions for the visceromotor regions, and the allostatic processes elicited by the latter would serve to fulfill these higher beliefs of self-efficacy. Conversely, beliefs about allostatic self-efficacy are updated by prediction errors communicated from the visceromotor regions.

Notably, while this type of metacognitive mechanism remains to be established for interoception and allostasis, it has been shown for other domains of cognition, including low-level processes such as visual discrimination performance (Zacharopoulos et al., 2014), that a prior belief of mastery enhances the actual performance (for reviews, see Bandura, 1977, 1989). Additionally, the proposed generalization of perceived low allostatic self-efficacy as a condition for the development from fatigue to depression requires that beliefs about allostatic mastery be broadcast beyond the circuit in Figure 7—for example, to areas involved in metacognition about other cognitive processes or circuits involved in regulation of mood—a process that should be detectable via differential effective connectivity of regions involved in metacognition about interoception and allostasis.

An alternative is that the metacognitive system not only receives prediction error signals from the visceromotor layer but has access to all levels in the hierarchy and monitors the performance of the interoceptive circuit as a whole, without influencing it. Since this circuit represents a generative model (of viscerosensory inputs), its performance or goodness would be indicated by the log evidence for the entire circuit (i.e., the cumulative negative surprise across all levels). A key question here is over what time window (into the past) this assessment takes place. Given a chosen time window, accumulated log evidence could be approximated by the integral of free energy, a quantity known as “free action” (Friston K. et al., 2010).

Turning to the neuroanatomy of metacognition, possible anatomical substrates have been investigated for several cognitive domains, in particular (extero)perceptual performance or memory (Shimamura and Squire, 1986; Schnyer et al., 2004; Fleming et al., 2010, 2012, 2014; McCurdy et al., 2013), but not, to our knowledge, for interoception or homeostasis/allostasis. Studies explicitly focused on metacognition of interoception are largely restricted to behavior (Garfinkel et al., 2015), with few neurophysiological investigations (but see Canales-Johnson et al., 2015). For other domains of cognition, such as exteroception or memory, the anterior prefrontal cortex (roughly corresponding to Brodmann's area 10) has been identified as a key area for metacognition by several neuroimaging and lesion studies (for review, see Fleming and Dolan, 2012). While the exact evaluative or monitoring mechanisms this region may perform are not well understood, the individual capacity for metacognition (of perceptual decision-making and memory) is reflected by functional connectivity (Baird et al., 2013).

By contrast, the involvement of anterior prefrontal cortex in metacognition of interoception has, to our knowledge, received little if any attention to date. Two empirical findings indicate that anterior prefrontal cortex is not an entirely implausible candidate region. First, it is known to exhibit functional connections with all key viscerosensory and visceromotor cortical regions of the circuit in Figure 7 (Baird et al., 2013). Second, tract tracing studies in the monkey demonstrated the existence of many of the structural connections implied by the first option described above, including direct (and largely reciprocal) connections from AIC, ACC and SGC to medial prefrontal pole (area 10 m; Carmichael and Price, 1996). Further evidence for anatomical connections between anterior prefrontal cortex and ACC as well as OFC was provided by human diffusion-weighted imaging (Liu et al., 2013).

Alternatively, the metacognitive layer may be represented within one of the visceromotor regions such as AIC or ACC; more specifically, within the hierarchically highest of their various subfields (cf. Carmichael and Price, 1996). For the ACC in particular, this possibility draws support from neuroimaging investigations that have provided evidence for a role of ACC in metacognitive functions such as performance monitoring and conflict detection (Carter et al., 1998; Botvinick et al., 1999).

### Empirical support for the hypothesis and future tests of its predictions

Above, we described our central clinical hypothesis with regard to the pathogenesis of fatigue and depression. In brief, we outlined how fatigue can be seen as an initial adaptive response to the metacognitive diagnosis of low allostatic self-efficacy; and how the chronic experience of dyshomeostasis may trigger a second phase in which beliefs about low self-efficacy generalize, inducing an abstract sense of lack of control and an all-encompassing sense of hopelessness. While direct tests of key predictions from this hypothesis remain to be performed, some empirical data support the plausibility of our proposal.

First, various studies indicate that the expression of fatigue and depression are associated with lesions or impairments of areas from our circuit model. For multiple sclerosis, (Hanken et al., 2014) reviewed neuroimaging studies relating fatigue to structural and functional properties of insula, ACC, and hypothalamus^6^. Additionally, neuropathological studies reported a high proportion of patients with inflammatory and demyelinating lesions of the hypothalamus, with indices of altered HPA activity (Huitinga et al., 2001, 2004). Most convincingly, neuropathological work focusing on cortex showed that in multiple sclerosis gray matter lesions are present throughout the entire cortex, but particularly frequently in cingulate and insular cortex (Haider et al., 2016). Concerning depression, Avery et al. (2014) examined non-medicated patients with fMRI and found that mid-insula activity during an interoceptive attention task was negatively correlated with the severity of depression and somatic symptoms; additionally functional connectivity during unconstrained cognition (rest) between mid-insula and SGC, OFC and amygdala was increased in patients and correlated with depression severity. Beyond the insula, neuroimaging has long identified SGC as a candidate site of primary pathophysiological importance in depression (Drevets et al., 1997; Mayberg et al., 1999). This region has a key role for inhibiting the amygdala and the sympathetic nervous system (Gold, 2015), which may be compromised by inflammatory processes (Miller and Raison, 2016).

Evidence from interventional studies is particularly worth noting. For example, in several elegant studies using MRI and PET in conjunction with typhoid vaccination to induce a peripheral immunological response and inflammation, Harrison and colleagues provided compelling evidence for structural and functional changes in posterior insula, anterior insula, and ACC (Harrison et al., 2009a,b, 2015). Importantly, they showed that inflammation-induced activity changes in posterior insula and ACC were associated with subjectively perceived fatigue, while activity changes in SGC predicted mood changes. Additionally, an fMRI study of patients receiving interferon-α treatment for hepatitis reported an abnormal increase in ACC activity during visuo-spatial attention (Capuron et al., 2005).

Second, clinical studies have demonstrated a striking link between fatigue and the occurrence of dyshomeostasis-inducing autonomic nervous system disorders (e.g., Stewart, 2000). Specifically, in MS patients, various measures of autonomic dysfunction correlate strongly with individual fatigue levels (Flachenecker et al., 2003; Cortez et al., 2015). However, to our knowledge, none of these studies examined metacognition about interoception or homeostasis/allostasis. Maher-Edwards et al. (2011) showed that metacognitive factors (including need for control of thoughts) predict individual levels of fatigue symptoms in CFS; however, the metacognitive assessment did not specifically consider interoception. Delgado-Pastor et al. (2015) did focus on metacognition of interoception and showed that increasing metacognitive abilities about interoception (by mindfulness-based interoceptive training) reduced worry more than increasing metacognition about other cognitive processes; however, this study did not specifically examine fatigue.

Generally, research on metacognition of interoception and homeostasis/allostasis has been relatively sparse so far (but see Khalsa et al., 2008; Garfinkel et al., 2015), and our hypothesis will require testing by specifically designed future studies. These studies will need to span four domains: behavioral-physiological studies that (i) confirm the proposed mediating role of metacognition in the link between dyshomeostasis and fatigue/depression; and computational neuroimaging studies that (ii) verify the operation of hierarchical Bayesian principles in interoceptive circuitry, (iii) demonstrate the plausibility of a metacognitive layer on top of the established circuits for homeostatic control, and (iv) demonstrate the existence of subgroups of patients in which the expression of fatigue and depression is predicted by a disturbance in either the afferent (interoceptive), efferent (control) or metacognitive branches of this system.

Importantly, testing the last three implications of our hypothesis requires mathematical models that can infer, from individual neurophysiological data, trial-wise precision-weighted predictions and prediction errors about viscerosensory inputs and how they dynamically alter connection strengths in interoceptive circuits—while respecting the layer-specific patterns of ascending (prediction errors) and descending (predictions) connections in cortical hierarchies (cf. Friston, 2008; Feldman-Barrett and Simmons, 2015). This brings us to analyses of functional and effective connectivity and methodological extensions of existing methods that are required to test our hypotheses.

## Extending models of effective connectivity

Functional connectivity refers to statistical dependencies between neurophysiological timeseries. It can be indexed by numerous statistical approaches, e.g., correlation analysis, autoregressive models, principal or independent component analysis (PCA, ICA) (Friston, 2011). Although advanced measures of functional connectivity can unearth directed influences (Friston et al., 2013; Seth et al., 2013), by itself functional connectivity does not disclose the mechanisms by which the measured signals were caused and may be vulnerable to confounds at the measurement level.

By contrast, other approaches are based on a forward model from hidden brain states to experimental measurements. These models do not strive for statistical characterizations of the data, but try to disambiguate alternative explanations of the data. Here, the focus is on effective connectivity, i.e., the “experiment- and time-dependent, simplest possible circuit diagram that would replicate the observed timing relationships between recorded neurons” (Aertsen and Preißl, 1999).

One approach to effective connectivity is provided by biophysical network models (BNMs; Honey et al., 2007; Jirsa et al., 2010; Woolrich and Stephan, 2013). BNMs consist of numerous (typically 10^2^–10^3^) neuronal network nodes, each of which is represented by a neural mass or mean field model of local neuronal populations. These nodes are connected by anatomical long-range connections (often informed by diffusion-weighted imaging data), and the resulting network activity is translated into node-specific measurements through an observation model. While their biological level of detail is attractive, a major limitation of BNMs is that their high degree of complexity renders the estimation of connection-specific parameters challenging (for review, see Stephan et al., 2015). Present BNMs only allow for a very limited number of parameters to be estimated, e.g., a single global scaling factor of connection strength (Deco et al., 2013). By contrast, testing our hypotheses requires models that provide fine-grained inference on different connections in hierarchical circuits for interoception and allostatic control (e.g., signaling of prediction errors along ascending connections originating in supragranular layers). In the future, this may be overcome by ongoing efforts to turn BNMs into fully generative models, with priors for different types of parameters, and importing advanced methods for model inversion from other approaches (for a discussion of this trend, Deco and Kringelbach, 2014; Stephan et al., 2015).

Fortunately, fully generative models are already available which fulfill many (albeit not all) of the requirements for testing the implications of our hypothesis, for example, dynamic causal modeling (DCM). Introduced in 2003 for fMRI data (Friston et al., 2003), DCM rests on a state space formulation and partitions the likelihood function (forward model) into two hierarchically related layers: while bilinear differential equations describe the dynamics of hidden (unobservable) interacting neuronal populations, a static observation equation transforms the ensuing mass activity of each population separately into a measurable signal. Inverting this model allows for inference on the effective connectivity among regions of interest, and how it is modulated by experimentally controlled conditions. Subsequent extensions have considered non-linear (Stephan et al., 2008) and stochastic differential equations (Li et al., 2011), which enable DCM to account for dynamic gain effects at synapses and intrinsic fluctuations, respectively. Similarly, extensions to the frequency domain allow for inference on connectivity from measurements during unconstrained cognition (also known as “resting state”) (Friston et al., 2014a).

The bilinear terms in DCM for fMRI allow for representing trial-wise modulation of connection strengths; this makes it well-suited for studying how connection strengths vary as a function of trial-by-trial prediction errors, where the latter are typically derived from a separate model applied to behavior or stimuli (e.g., den Ouden et al., 2010). However, extending this to interoceptive prediction errors and their role in hierarchically structured circuits faces several non-trivial challenges. First, while it is easy to induce prediction errors in exteroceptive paradigms, this is less trivial in the interoceptive domain, particularly in a way that is non-invasive and patient-friendly. With the exception of manipulations of inspiratory breathing load (Paulus et al., 2012), we presently lack non-invasive methods to do so, and new paradigms will need to be developed. An alternative option is to extract prediction errors from naturally occurring irregularities in bodily rhythms (e.g., variations in heartbeat intervals); this will be presented in future work. Second, existing formulations of DCM only consider a coarse representation of neuronal populations and do not, for example, differentiate between different layers and layer-specific connections. Thus, they lack the anatomical specificity required to fully test the above predictions. With the advent of high-field MRI, it is now possible, in principle, to obtain sufficiently high resolution that separate cortical layers can be imaged in humans (e.g., Koopmans et al., 2010; Olman et al., 2012). For example, consistent with predictive coding, a recent study was able to decode contextual information from superficial laminae of parts of primary visual cortex that did not receive direct “bottom up” input but which plausibly received top-down predictions from hierarchically higher regions (Muckli et al., 2015).

However, signals in upper cortical layers are contaminated by blood draining effects from lower layers. This confounds the identification of layer-specific activity and connections and requires adapting generative models of fMRI data. While a first model was recently developed to account for these layer-specific hemodynamic effects (Heinzle et al., 2016), this is so far restricted to the level of a single region, and further work is required to extend this to a network-level DCM.

DCM has also been formulated for M/EEG data, serving to explain a variety of data features such as event-related potentials (David et al., 2006), induced responses (Chen et al., 2008), or steady-state responses (Moran et al., 2009). The rich temporal information in M/EEG data allows for modeling far more detailed circuit architectures than DCM for fMRI. Specifically, DCM for M/EEG considers columnar cortical units which consist of different types of neurons (pyramidal cells, excitatory and inhibitory interneurons) and communicate through synaptic connections with laminar specificity. This allows for differentiating between the different type of connections (ascending and descending) in cortical hierarchies, as required to test for specific effects of predictions and prediction errors. However, existing formulations of DCM for M/EEG are fitted to averaged data (e.g., event-related potentials) and only consider modulatory effects across different experimental conditions. To test our hypotheses, extensions are needed which account for trial-wise prediction error effects on connections. The poor signal-to-noise ratio of single-trial recordings poses a serious challenge for modeling (Brodersen et al., 2011) and may require adapting hierarchical (empirical Bayesian) estimation schemes (Friston et al., 2016; Raman et al., 2016) to single-trial scenaria as well as sampling schemes for model inversion in DCM; the computational costs of the latter may require moving to GPU-based numerical schemes (Aponte et al., 2016).

A third extension of generative models could move beyond the current formulations of DCM altogether and consider models that are less directly connected to physiology, but are capable of modeling perceptual inference within trials and learning (belief updates) across trials. This could encompass generative models of trial-wise M/EEG responses where, for example, trial by trial amplitudes are predicted as a linear mixture of prediction errors (Lieder et al., 2013). Alternatively, hierarchically structured predictive coding circuits (Friston, 2005; Bogacz, in press) could be used to analyse trial-wise electrophysiological data, allowing for a closer connection to physiology.

In summary, while existing generative models of neuroimaging data provide a crucial platform for testing our hypothesis, no existing model fully meets the requirements and several extensions will be required.

## Differential diagnosis of fatigue and depression

If the key predictions from our hypothesis are found to be correct and if a generative model of neuroimaging or electrophysiological measurements of the interoceptive-allostatic circuit in Figure 7 could be established, this might have important implications for the clinical management of fatigue and depression, in particular differential diagnosis. Specifically, comparing alternative models of effective connectivity could help disambiguate different origins of circuit dysfunction leading to fatigue and depression, respectively.

The circuit model displayed by Figure 7 highlights that fatigue could result from functional disturbances or structural lesions—such as local inflammatory or demyelinating processes—in very different locations. According to our circuit model, the experience of chronic dyshomeostasis may be due to an initial pathology at the level of:

- “sensors” (viscerosensory areas)—corresponding to the “illusion” of dyshomeostasis,
- “allostatic predictors” (visceromotor regions)—equivalent to flawed predictions of bodily states,
- “effector” regions (hypothalamus, brainstem, spinal cord)—that is, at the level of homeostatic reflex arcs,
- in the body itself—for example, a disease process that evades attempts of homeostatic regulation by (at least initially) intact cerebral circuits (e.g., autoimmunological processes or cancer),
- at the metacognitive level—in this case, insufficient regulation of bodily states would be the consequence, not the cause, of beliefs about low allostatic self-efficacy.

This suggests that patients with fatigue and depression, respectively, could be classified into several subgroups that differ in terms of the location as well as the type of disease mechanism. While a disturbance at any of the above locations will lead to compensatory changes throughout the entire circuit, the resulting patterns of effective connectivity might be distinguishable, particularly in the context of homeostatic perturbations. Notably, in internal medicine, differential diagnosis with regard to compensatory changes in feedback circuits is commonplace, such as distinguishing metabolic and respiratory origins of acidosis, or identifying hypothalamic, pituitary or peripheral causes for endocrine dysfunction. In our approach, such differential diagnosis could be implemented formally by model selection, i.e., evaluating the evidence of different models that assume a disturbance at different branches and are fitted to electrophysiological or fMRI data from an individual patient (cf. Stephan et al., 2016a). We will examine this possibility in future work, simulating circuit activity under different types of lesions and different perturbations^7^.

The first of the cases described above deserves special consideration: when there is initially no real state of dyshomeostasis, but dyshomeostasis is only subjectively perceived due to damage to viscerosensory pathways. For example, in MS, lesions frequently affect insular cortex (Haider et al., 2016); this “broken sensor” would create PE signals (interoceptive surprise) that would be interpreted by visceromotor regions as bodily dyshomeostasis. As the emitted control actions cannot reduce interoceptive surprise, a metacognitive interpretation ensues that leads to the subjective feeling of fatigue, as discussed above. This case of an “illusion” of dyshomeostasis illustrates that fatigue is always an interpretation of perceived (not necessarily real) dyshomeostasis. This may apply beyond interoception in that fatigue could also result from other forms of surprise that are not reduced by adequate actions. For example, brain damage outside interoceptive pathways can invoke general changes in performance levels, for example, slowing of cognitive and motor acts due to demyelination and hence reduced conduction speed in MS. The metacognitive detection of such a general slowing of cognition and action, and the experience that adequate actions (in this case, rest) do not reduce surprise about performance levels (metacognitive surprise), may lead to a similar sensation of fatigue as when caused by bodily dyshomeostasis. This suggests that when primary brain diseases do not impair interoception or allostatic control (e.g., cases of stroke or MS outside viscerosensory/visceromotor regions) may also induce a subjective sensation of fatigue by means of a metacognitive mechanism.

An additional possible reason for dysfunction of the interoceptive-allostatic circuit must be highlighted: aberrant neuromodulatory input. Monoaminergic brain stem nuclei are in receipt of viscerosensory inputs and project to many, if not all, components of the interoceptive-allostatic circuit in Figure 7 (Craig, 2003; Critchley and Harrison, 2013). There is now considerable evidence that one possible cause of fatigue is an impairment of these monoaminergic brainstem projections with reduced availability of dopamine, serotonin and noradrenaline at their (sub)cortical target sites (for review, see Dantzer et al., 2014). This can be caused by inflammatory processes—not only of intra-cerebral origin, but also due to chronic peripheral inflammatory processes which, through well understood biochemical cascades, lead to reduced synthesis of dopamine, serotonin and noradrenaline in brainstem neurons (Dantzer et al., 2014). In the context of the theory proposed in this paper, a reduced dopaminergic supply in particular may impact on the precision ratio which governs the weighting of prediction errors (compare Equations 3, 6). This is because various neurophysiological studies in humans and animals indicate that one of the computational quantities encoded by variations in dopamine release is uncertainty (inverse precision) (Fiorillo et al., 2003; de Lafuente and Romo, 2011; Hart et al., 2015; Schwartenbeck et al., 2015a; Tomassini et al., 2016). Notably, many if not all regions of the interoceptive circuit in Figure 7, are characterized by a high density of dopaminergic receptors and terminals across all cortical layers; this is particularly well-established for visceromotor regions like AIC, ACC, or OFC (Gaspar et al., 1989; Hurd et al., 2001; Lewis et al., 2001). The role of dopamine for viscerosensory (posterior insula) regions is less well established but *in situ* hybridisation studies point to the existence of dopamine receptor mRNA in human posterior insula as well (Hurd et al., 2001).

An interesting corollary of our hypothesis is that, in principle, the chronic disturbance of *any* homeostatically critical physiological variable *in any direction* has the potential of inducing fatigue and depression. This is consistent with the fact that chronic diseases of very different nature that do not directly affect the brain are frequently accompanied by fatigue and depression (e.g., hepatitis, cancer, diabetes, fibromyalgia)—and may explain the counterintuitive observation that this includes endocrine and metabolic disorders which enhance (rather than decrease) the metabolic availability of energy und the activation/excitability levels of numerous tissues, e.g., hyperthyrodism, Cushing's syndrome, or hypercalcemia (Kaltsas et al., 2010).

Finally, while the present work has focused exclusively on interoception and bodily homeostasis/allostasis, it may be seen as a prelude to a wider concept of what one might call “generalized allostasis”: the active inference notion that humans have setpoints (hold beliefs) with regard to many aspects of the physical, social and cognitive world; that they try to reach these setpoints (fulfill these beliefs) by adequate actions; and that they can, in principle, prospectively adjust these setpoints in order to elicit actions. Here, one key issue is that reaching one specific setpoint may compromise one's ability to reach another. For example, holding negative beliefs about states of the world (cf. “depressive realism”; Alloy and Abramson, 1988) could be seen as an allostatic change of setpoint that renders bad outcomes expected and should therefore lead to future homeostasis. However, this may come at the cost of violating higher setpoints, such as a belief that one expects to have a certain capacity for control, or that protective forces should exist in the world (e.g., caring other agents). Similarly, ensuring one's own bodily homeostasis can conflict with beliefs about other aspects of the world, and there are ample empirical demonstrations of humans' willingness to forego bodily homeostasis and sacrifice themselves in order to fulfill beliefs that transcend their own existence—for example, beliefs that loved ones should be protected or that certain religious principles must be upheld. This raises the interesting question what, ultimately, the highest setpoint or belief is that dictates the behavior of individual humans.

## Conclusions

This paper contains three main contributions. First, we revisited how traditional homeostatic concepts can be merged with Bayesian perspectives on interoception, leading to formal definitions for dyshomeostasis (chronically enhanced interoceptive surprise, or, equivalently, low evidence for the brain's generative model of viscerosensory inputs) and allostasis (the change in prior beliefs which define setpoints of homeostatic reflex arcs). Second, these definitions allowed for a bridge to metacognition and the postulate that the performance of the interoceptive circuit is being monitored by a higher metacognitive layer, possibly located in anterior prefrontal cortex, which encodes and updates beliefs about the brain's capacity to successfully regulate bodily states (allostatic self-efficacy). Third, we suggested a two-stage process where fatigue might represent an initial adaptive response to the metacognitive diagnosis of low allostatic self-efficacy, while the enduring experience of dyshomeostasis may initiate a second phase in which low self-efficacy beliefs generalize, leading to an all-encompassing sense of lack of control and hopelessness.

The perspective offered by this paper may be useful to further our understanding of the pathogenesis of fatigue, and how it may be understood as a high-level interpretation of the brain in monitoring its own efforts to control a vital part of its environment, the body. We hope that this theoretical framework and the methodological extensions of models of effective connectivity it suggests will eventually lead to applications of diagnostic utility, in particular, for stratifying patients from spectrum diseases in whom fatigue and hopelessness are leading symptoms, such as multiple sclerosis or depression.

## Author contributions

Conceptual discussions: KS, ZM, CM, LW, SP, TG, MT, SF, HH, AS, and FP. Derived mathematical theory: KS, CM. Implemented the simulations: KS. Wrote the manuscript: KS, ZM, CM, LW, SP, TG, MT, SF, HH, AS, and FP. Figures: HH and KS.

### Conflict of interest statement

The handling Editor declared a shared affiliation, though no other collaboration, with several of the authors KS, CM, SF and states that the process nevertheless met the standards of a fair and objective review. The other authors declare that the research was conducted in the absence of any commercial or financial relationships that could be construed as a potential conflict of interest.

## Footnotes

^1^In the well-known framework by Marr (1982), these levels are referred to as the “computational,” “algorithmic,” and “implementational” levels of analysis, respectively. Considering the original definition of “computation” in theoretical computer science, these designations are partially confusing, and in the following we will therefore refer to the first level as the “teleological” (purpose) level of description.

^2^Below, we will define homeostatic setpoints as the expectations (means) of prior beliefs about the states the body should inhabit, and homeostatic range as the variance of these prior beliefs.

^3^For example, eye movements do not influence the environment beyond the body (social interactions perhaps excepted), but determine sampling of visual information.

^4^Here, we are pragmatically changing the notation of time (from index to argument) as we find it more natural to express the action signal in continuous time.

^5^In principle, of any sort—although clearly some viscerosensory domains (e.g., about cardiac function) may exert greater impact on self-efficacy than others.

^6^We would like to add the cautionary note that in MS research the literature on MRI-based morphometric studies shows striking variability (see Popescu et al., 2016).

^7^A separate potential subgroup is worth mentioning that also relates to a concept of homeostasis but is distinct from our hypothesis. In some patients, fatigue and/or depression may not result from a metacognitive reaction to a (perceived or real) chronic state of dyshomeostasis, as our hypothesis states, but represent the result of fulfilling a high-order belief (and thus represent a state of homeostasis)—for example, that one deserves a socially inferior position along with exhaustion and sadness.

## References

[B1] Abramson L. Y. Seligman M. E. Teasdale J. D. (1978). Learned helplessness in humans: critique and reformulation. J. Abnorm. Psychol. 87, 49–74. 10.1037/0021-843X.87.1.49 649856

[B2] Adams R. A. Perrinet L. U. Friston K. (2012). Smooth pursuit and visual occlusion: active inference and oculomotor control in schizophrenia. PLoS ONE 7:e47502. 10.1371/journal.pone.0047502 23110076 PMC3482214

[B3] Adams R. A. Shipp S. Friston K. J. (2013a). Predictions not commands: active inference in the motor system. Brain Struct. Funct. 218, 611–643. 10.1007/s00429-012-0475-5 23129312 PMC3637647

[B4] Adams R. A. Stephan K. E. Brown H. R. Frith C. D. Friston K. J. (2013b). The computational anatomy of psychosis. Front. Psychiatry 4:47. 10.3389/fpsyt.2013.00047 23750138 PMC3667557

[B5] Aertsen A. Preißl H. (1999). Dynamics of activity and connectivity in physiological neuronal networks, in Nonlinear Dynamics and Neuronal Networks, ed Schuster H. (New York, NY: Schuster VCH Publishers), 281–302.

[B6] Alink A. Schwiedrzik C. M. Kohler A. Singer W. Muckli L. (2010). Stimulus predictability reduces responses in primary visual cortex. J. Neurosci. 30, 2960–2966. 10.1523/JNEUROSCI.3730-10.2010 20181593 PMC6633950

[B7] Allen G. V. Saper C. B. Hurley K. M. Cechetto D. F. (1991). Organization of visceral and limbic connections in the insular cortex of the rat. J. Comp. Neurol. 311, 1–16. 10.1002/cne.903110102 1719041

[B8] Alloy L. B. Abramson L. Y. (1988). Depressive realism: four theoretical perspectives, in Cognitive Processes in Depression, ed Alloy L. B. (New York, NY: Guilford Press), 223–265.

[B9] Andolina I. M. Jones H. E. Sillito A. M. (2013). Effects of cortical feedback on the spatial properties of relay cells in the lateral geniculate nucleus. J. Neurophysiol. 109, 889–899. 10.1152/jn.00194.2012 23100142 PMC3567383

[B10] Angelucci A. Bressloff P. C. (2006). Contribution of feedforward, lateral and feedback connections to the classical receptive field center and extra-classical receptive field surround of primate V1 neurons. Prog. Brain Res. 154, 93–120. 10.1016/S0079-6123(06)54005-1 17010705

[B11] Aponte E. A. Raman S. Sengupta B. Penny W. D. Stephan K. E. Heinzle J. (2016). mpdcm: a toolbox for massively parallel dynamic causal modeling. J. Neurosci. Methods 257, 7–16. 10.1016/j.jneumeth.2015.09.009 26384541

[B12] Arnstein P. Caudill M. Mandle C. L. Norris A. Beasley R. (1999). Self efficacy as a mediator of the relationship between pain intensity, disability and depression in chronic pain patients. Pain 80, 483–491. 10.1016/S0304-3959(98)00220-6 10342410

[B13] Ashby W. R. (1954). Design for a Brain. New York, NY: John Wiley & Sons.

[B14] Ashby W. R. (1956). An Introduction to Cybernetics. London: Chapman & Hall.

[B15] Avery J. A. Drevets W. C. Moseman S. E. Bodurka J. Barcalow J. C. Simmons W. K. (2014). Major depressive disorder is associated with abnormal interoceptive activity and functional connectivity in the insula. Biol. Psychiatry 76, 258–266. 10.1016/j.biopsych.2013.11.027 24387823 PMC4048794

[B16] Baird B. Smallwood J. Gorgolewski K. J. Margulies D. S. (2013). Medial and lateral networks in anterior prefrontal cortex support metacognitive ability for memory and perception. J. Neurosci. 33, 16657–16665. 10.1523/JNEUROSCI.0786-13.2013 24133268 PMC6618531

[B17] Bakshi R. Shaikh Z. A. Miletich R. S. Czarnecki D. Dmochowski J. Henschel K.. (2000). Fatigue in multiple sclerosis and its relationship to depression and neurologic disability. Mult. Scler. 6, 181–185. 10.1177/135245850000600308 10871830

[B18] Bandura A. (1977). Self-efficacy: toward a unifying theory of behavioral change. Psychol. Rev. 84, 191–215. 10.1037/0033-295X.84.2.191 847061

[B19] Bandura A. (1989). Human agency in social cognitive theory. Am. Psychol. 44, 1175–1184. 10.1037/0003-066X.44.9.1175 2782727

[B20] Bandura A. Barbaranelli C. Caprara G. V. Pastorelli C. (1996). Multifaceted impact of self-efficacy beliefs on academic functioning. Child Dev. 67, 1206–1222. 10.2307/1131888 8706518

[B21] Bandura A. O'Leary A. Taylor C. B. Gauthier J. Gossard D. (1987). Perceived self-efficacy and pain control: opioid and nonopioid mechanisms. J. Pers. Soc. Psychol. 53, 563–571. 10.1037/0022-3514.53.3.563 2821217

[B22] Bandura A. Pastorelli C. Barbaranelli C. Caprara G. V. (1999). Self-efficacy pathways to childhood depression. J. Pers. Soc. Psychol. 76, 258–269. 10.1037/0022-3514.76.2.258 10074708

[B23] Bastos A. M. Usrey W. M. Adams R. A. Mangun G. R. Fries P. Friston K. J. (2012). Canonical microcircuits for predictive coding. Neuron 76, 695–711. 10.1016/j.neuron.2012.10.038 23177956 PMC3777738

[B24] Behrens T. E. Woolrich M. W. Walton M. E. Rushworth M. F. (2007). Learning the value of information in an uncertain world. Nat. Neurosci. 10, 1214–1221. 10.1038/nn1954 17676057

[B25] Bogacz R. (in press). A tutorial on the free-energy framework for modelling perception learning. J. Math. Psychol. 10.1016/j.jmp.2015.11.003. PMC5341759 28298703

[B26] Botvinick M. Nystrom L. E. Fissell K. Carter C. S. Cohen J. D. (1999). Conflict monitoring versus selection-for-action in anterior cingulate cortex. Nature 402, 179–181. 10.1038/46035 10647008

[B27] Breakspear M. Roberts G. Green M. J. Nguyen V. T. Frankland A. Levy F.. (2015). Network dysfunction of emotional and cognitive processes in those at genetic risk of bipolar disorder. Brain 138, 3427–3439. 10.1093/brain/awv261 26373604

[B28] Brodersen K. H. Haiss F. Ong C. S. Jung F. Tittgemeyer M. Buhmann J. M.. (2011). Model-based feature construction for multivariate decoding. Neuroimage 56, 601–615. 10.1016/j.neuroimage.2010.04.036 20406688 PMC3112410

[B29] Burke A. L. Mathias J. L. Denson L. A. (2015). Psychological functioning of people living with chronic pain: a meta-analytic review. Br. J. Clin. Psychol. 54, 345–360. 10.1111/bjc.12078 25772553

[B30] Canales-Johnson A. Silva C. Huepe D. Rivera-Rei A. Noreika V. Garcia Mdel C.. (2015). Auditory feedback differentially modulates behavioral and neural markers of objective and subjective performance when tapping to your heartbeat. Cereb. Cortex 25, 4490–4503. 10.1093/cercor/bhv076 25899708 PMC4816795

[B31] Cannon W. B. (1929). Organization for physiological homeostasis. Physiol. Rev. 9, 399–431.

[B32] Capuron L. Pagnoni G. Demetrashvili M. Woolwine B. J. Nemeroff C. B. Berns G. S.. (2005). Anterior cingulate activation and error processing during interferon-alpha treatment. Biol. Psychiatry 58, 190–196. 10.1016/j.biopsych.2005.03.033 16084839 PMC1366492

[B33] Carmichael S. T. Price J. L. (1995). Limbic connections of the orbital and medial prefrontal cortex in macaque monkeys. J. Comp. Neurol. 363, 615–641. 10.1002/cne.903630408 8847421

[B34] Carmichael S. T. Price J. L. (1996). Connectional networks within the orbital and medial prefrontal cortex of macaque monkeys. J. Comp. Neurol. 371, 179–207. 8835726 10.1002/(SICI)1096-9861(19960722)371:2<179::AID-CNE1>3.0.CO;2-#

[B35] Carter C. S. Braver T. S. Barch D. M. Botvinick M. M. Noll D. Cohen J. D. (1998). Anterior cingulate cortex, error detection, and the online monitoring of performance. Science 280, 747–749. 10.1126/science.280.5364.747 9563953

[B36] Carver C. S. Scheier M. F. (1982). Control theory: a useful conceptual framework for personality-social, clinical, and health psychology. Psychol. Bull. 92, 111–135. 10.1037/0033-2909.92.1.111 7134324

[B37] Cechetto D. F. Saper C. B. (1987). Evidence for a viscerotopic sensory representation in the cortex and thalamus in the rat. J. Comp. Neurol. 262, 27–45. 10.1002/cne.902620104 2442207

[B38] Chaudhuri A. Behan P. O. (2004). Fatigue in neurological disorders. Lancet 363, 978–988. 10.1016/S0140-6736(04)15794-2 15043967

[B39] Chen C. C. Kiebel S. J. Friston K. J. (2008). Dynamic causal modelling of induced responses. Neuroimage 41, 1293–1312. 10.1016/j.neuroimage.2008.03.026 18485744

[B40] Chiba T. Kayahara T. Nakano K. (2001). Efferent projections of infralimbic and prelimbic areas of the medial prefrontal cortex in the Japanese monkey, Macaca fuscata. Brain Res. 888, 83–101. 10.1016/S0006-8993(00)03013-4 11146055

[B41] Clark I. Dumas G. (2015). The regulation of task performance: a trans-disciplinary review. Front. Psychol. 6:1862. 10.3389/fpsyg.2015.01862 26779050 PMC4703823

[B42] Conant R. C. Ashby W. R. (1970). Every good regulator of a system must be a model of that system. Int. J. Syst. Sci. 1, 89–97. 10.1080/00207727008920220

[B43] Cooray G. K. Sengupta B. Douglas P. Englund M. Wickstrom R. Friston K. (2015). Characterising seizures in anti-NMDA-receptor encephalitis with dynamic causal modelling. Neuroimage 118, 508–519. 10.1016/j.neuroimage.2015.05.064 26032883 PMC4558461

[B44] Corlett P. R. Frith C. D. Fletcher P. C. (2009). From drugs to deprivation: a Bayesian framework for understanding models of psychosis. Psychopharmacology (Berl). 206, 515–530. 10.1007/s00213-009-1561-0 19475401 PMC2755113

[B45] Corlett P. R. Taylor J. R. Wang X. J. Fletcher P. C. Krystal J. H. (2010). Toward a neurobiology of delusions. Prog. Neurobiol. 92, 345–369. 10.1016/j.pneurobio.2010.06.007 20558235 PMC3676875

[B46] Cortez M. M. Nagi Reddy S. K. Goodman B. Carter J. L. Wingerchuk D. M. (2015). Autonomic symptom burden is associated with MS-related fatigue and quality of life. Mult. Scler. Relat. Disord. 4, 258–263. 10.1016/j.msard.2015.03.007 26008943

[B47] Craig A. D. (2002). How do you feel? Interoception: the sense of the physiological condition of the body. Nat. Rev. Neurosci. 3, 655–666. 10.1038/nrn894 12154366

[B48] Craig A. D. (2003). Interoception: the sense of the physiological condition of the body. Curr. Opin. Neurobiol. 13, 500–505. 10.1016/S0959-4388(03)00090-4 12965300

[B49] Critchley H. D. Harrison N. A. (2013). Visceral influences on brain and behavior. Neuron 77, 624–638. 10.1016/j.neuron.2013.02.008 23439117

[B50] Dantzer R. Heijnen C. J. Kavelaars A. Laye S. Capuron L. (2014). The neuroimmune basis of fatigue. Trends Neurosci. 37, 39–46. 10.1016/j.tins.2013.10.003 24239063 PMC3889707

[B51] Dantzer R. Kelley K. W. (2007). Twenty years of research on cytokine-induced sickness behavior. Brain Behav. Immun. 21, 153–160. 10.1016/j.bbi.2006.09.006 17088043 PMC1850954

[B52] Daunizeau J. David O. Stephan K. E. (2011). Dynamic causal modelling: a critical review of the biophysical and statistical foundations. Neuroimage 58, 312–322. 10.1016/j.neuroimage.2009.11.062 19961941

[B53] Daunizeau J. den Ouden H. E. Pessiglione M. Kiebel S. J. Stephan K. E. Friston K. J. (2010). Observing the observer (I): meta-bayesian models of learning and decision-making. PLoS ONE 5:e15554. 10.1371/journal.pone.0015554 21179480 PMC3001878

[B54] David O. Kiebel S. J. Harrison L. M. Mattout J. Kilner J. M. Friston K. J. (2006). Dynamic causal modeling of evoked responses in EEG and MEG. Neuroimage 30, 1255–1272. 10.1016/j.neuroimage.2005.10.045 16473023

[B55] Dayan P. Daw N. D. (2008). Decision theory, reinforcement learning, and the brain. Cogn. Affect. Behav. Neurosci. 8, 429–453. 10.3758/CABN.8.4.429 19033240

[B56] Dayan P. Hinton G. E. Neal R. M. Zemel R. S. (1995). The Helmholtz machine. Neural Comput. 7, 889–904. 10.1162/neco.1995.7.5.889 7584891

[B57] Deco G. Kringelbach M. L. (2014). Great expectations: using whole-brain computational connectomics for understanding neuropsychiatric disorders. Neuron 84, 892–905. 10.1016/j.neuron.2014.08.034 25475184

[B58] Deco G. Ponce-Alvarez A. Mantini D. Romani G. L. Hagmann P. Corbetta M. (2013). Resting-state functional connectivity emerges from structurally and dynamically shaped slow linear fluctuations. J. Neurosci. 33, 11239–11252. 10.1523/JNEUROSCI.1091-13.2013 23825427 PMC3718368

[B59] de Lafuente V. Romo R. (2011). Dopamine neurons code subjective sensory experience and uncertainty of perceptual decisions. Proc. Natl. Acad. Sci. U.S.A. 108, 19767–19771. 10.1073/pnas.1117636108 22106310 PMC3241809

[B60] Delgado-Pastor L. C. Ciria L. F. Blanca B. Mata J. L. Vera M. N. Vila J. (2015). Dissociation between the cognitive and interoceptive components of mindfulness in the treatment of chronic worry. J. Behav. Ther. Exp. Psychiatry 48, 192–199. 10.1016/j.jbtep.2015.04.001 25912677

[B61] Denison E. Asenlof P. Lindberg P. (2004). Self-efficacy, fear avoidance, and pain intensity as predictors of disability in subacute and chronic musculoskeletal pain patients in primary health care. Pain 111, 245–252. 10.1016/j.pain.2004.07.001 15363867

[B62] den Ouden H. E. Daunizeau J. Roiser J. Friston K. J. Stephan K. E. (2010). Striatal prediction error modulates cortical coupling. J. Neurosci. 30, 3210–3219. 10.1523/JNEUROSCI.4458-09.2010 20203180 PMC3044875

[B63] Doya K. Ishii S. Pouget A. Rao R. P. (2011). Bayesian Brain: Probabilistic Approaches to Neural Coding. Cambridge, MA: MIT Press.

[B64] Drevets W. C. Price J. L. Simpson J. R. Jr. Todd R. D. Reich T. Vannier M.. (1997). Subgenual prefrontal cortex abnormalities in mood disorders. Nature 386, 824–827. 10.1038/386824a0 9126739

[B65] Feldman-Barrett L. F. Simmons W. K. (2015). Interoceptive predictions in the brain. Nat. Rev. Neurosci. 16, 419–429. 10.1038/nrn3950 26016744 PMC4731102

[B66] Felleman D. J. Van Essen D. C. (1991). Distributed hierarchical processing in the primate cerebral cortex. Cereb. Cortex 1, 1–47. 10.1093/cercor/1.1.1 1822724

[B67] Fiorillo C. D. Tobler P. N. Schultz W. (2003). Discrete coding of reward probability and uncertainty by dopamine neurons. Science 299, 1898–1902. 10.1126/science.1077349 12649484

[B68] Flachenecker P. Rufer A. Bihler I. Hippel C. Reiners K. Toyka K. V.. (2003). Fatigue in MS is related to sympathetic vasomotor dysfunction. Neurology 61, 851–853. 10.1212/01.WNL.0000080365.95436.B8 14504339

[B69] Fleming S. M. Dolan R. J. (2012). The neural basis of metacognitive ability. Philos. Trans. R. Soc. Lond. B. Biol. Sci. 367, 1338–1349. 10.1098/rstb.2011.0417 22492751 PMC3318765

[B70] Fleming S. M. Huijgen J. Dolan R. J. (2012). Prefrontal contributions to metacognition in perceptual decision making. J. Neurosci. 32, 6117–6125. 10.1523/JNEUROSCI.6489-11.2012 22553018 PMC3359781

[B71] Fleming S. M. Ryu J. Golfinos J. G. Blackmon K. E. (2014). Domain-specific impairment in metacognitive accuracy following anterior prefrontal lesions. Brain 137, 2811–2822. 10.1093/brain/awu221 25100039 PMC4163038

[B72] Fleming S. M. Weil R. S. Nagy Z. Dolan R. J. Rees G. (2010). Relating introspective accuracy to individual differences in brain structure. Science 329, 1541–1543. 10.1126/science.1191883 20847276 PMC3173849

[B73] Freedman L. J. Insel T. R. Smith Y. (2000). Subcortical projections of area 25 (subgenual cortex) of the macaque monkey. J. Comp. Neurol. 421, 172–188. 10.1002/(SICI)1096-9861(20000529)421:2<172::AID-CNE4>3.0.CO;2-8 10813780

[B74] Friston K. (2003). Learning and inference in the brain. Neural Netw. 16, 1325–1352. 10.1016/j.neunet.2003.06.005 14622888

[B75] Friston K. (2005). A theory of cortical responses. Philos. Trans. R. Soc. Lond. B. Biol. Sci. 360, 815–836. 10.1098/rstb.2005.1622 15937014 PMC1569488

[B76] Friston K. (2008). Hierarchical models in the brain. PLoS Comput. Biol. 4:e1000211. 10.1371/journal.pcbi.1000211 18989391 PMC2570625

[B77] Friston K. (2009). The free-energy principle: a rough guide to the brain? Trends Cogn. Sci. 13, 293–301. 10.1016/j.tics.2009.04.005 19559644

[B78] Friston K. (2010). The free-energy principle: a unified brain theory? Nat. Rev. Neurosci. 11, 127–138. 10.1038/nrn2787 20068583

[B79] Friston K. Adams R. Montague R. (2012). What is value-accumulated reward or evidence? Front. Neurorobot. 6:11. 10.3389/fnbot.2012.00011 23133414 PMC3487150

[B80] Friston K. J. (2011). Functional and effective connectivity: a review. Brain Connect. 1, 13–36. 10.1089/brain.2011.0008 22432952

[B81] Friston K. J. Daunizeau J. Kilner J. Kiebel S. J. (2010). Action and behavior: a free-energy formulation. Biol. Cybern. 102, 227–260. 10.1007/s00422-010-0364-z 20148260

[B82] Friston K. J. Harrison L. Penny W. (2003). Dynamic causal modelling. Neuroimage 19, 1273–1302. 10.1016/S1053-8119(03)00202-7 12948688

[B83] Friston K. J. Kahan J. Biswal B. Razi A. (2014a). A DCM for resting state fMRI. Neuroimage 94, 396–407. 10.1016/j.neuroimage.2013.12.009 24345387 PMC4073651

[B84] Friston K. J. Litvak V. Oswal A. Razi A. Stephan K. E. van Wijk B. C.. (2016). Bayesian model reduction and empirical Bayes for group (DCM) studies. Neuroimage 128, 413–431. 10.1016/j.neuroimage.2015.11.015 26569570 PMC4767224

[B85] Friston K. J. Stephan K. E. Montague R. Dolan R. J. (2014b). Computational psychiatry: the brain as a phantastic organ. Lancet Psychiatry 1, 148–158. 10.1016/S2215-0366(14)70275-5 26360579

[B86] Friston K. Kilner J. Harrison L. (2006). A free energy principle for the brain. J. Physiol. Paris 100, 70–87. 10.1016/j.jphysparis.2006.10.001 17097864

[B87] Friston K. Moran R. Seth A. K. (2013). Analysing connectivity with Granger causality and dynamic causal modelling. Curr. Opin. Neurobiol. 23, 172–178. 10.1016/j.conb.2012.11.010 23265964 PMC3925802

[B88] Friston K. Rigoli F. Ognibene D. Mathys C. Fitzgerald T. Pezzulo G. (2015). Active inference and epistemic value. Cogn. Neurosci. 6, 187–214. 10.1080/17588928.2015.1020053 25689102

[B89] Friston K. Stephan K. E. Li B. Daunizeau J. (2010). Generalised filtering. Math. Prob. Eng. 2010:621670 10.1155/2010/621670

[B90] Frith C. D. Frith U. (2012). Mechanisms of social cognition. Annu. Rev. Psychol. 63, 287–313. 10.1146/annurev-psych-120710-100449 21838544

[B91] Garfinkel S. N. Seth A. K. Barrett A. B. Suzuki K. Critchley H. D. (2015). Knowing your own heart: distinguishing interoceptive accuracy from interoceptive awareness. Biol. Psychol. 104, 65–74. 10.1016/j.biopsycho.2014.11.004 25451381

[B92] Garrido M. I. Friston K. J. Kiebel S. J. Stephan K. E. Baldeweg T. Kilner J. M. (2008). The functional anatomy of the MMN: a DCM study of the roving paradigm. Neuroimage 42, 936–944. 10.1016/j.neuroimage.2008.05.018 18602841 PMC2640481

[B93] Gaspar P. Berger B. Febvret A. Vigny A. Henry J. P. (1989). Catecholamine innervation of the human cerebral cortex as revealed by comparative immunohistochemistry of tyrosine hydroxylase and dopamine-beta-hydroxylase. J. Comp. Neurol. 279, 249–271. 10.1002/cne.902790208 2563268

[B94] Geisler W. S. Diehl R. L. (2002). Bayesian natural selection and the evolution of perceptual systems. Philos. Trans. R. Soc. Lond. B. Biol. Sci. 357, 419–448. 10.1098/rstb.2001.1055 12028784 PMC1692963

[B95] Geisler W. S. Kersten D. (2002). Illusions, perception and Bayes. Nat. Neurosci. 5, 508–510. 10.1038/nn0602-508 12037517

[B96] Gilbert J. R. Symmonds M. Hanna M. G. Dolan R. J. Friston K. J. Moran R. J. (2016). Profiling neuronal ion channelopathies with non-invasive brain imaging and dynamic causal models: case studies of single gene mutations. Neuroimage 124, 43–53. 10.1016/j.neuroimage.2015.08.057 26342528 PMC4655917

[B97] Gold P. W. (2015). The organization of the stress system and its dysregulation in depressive illness. Mol. Psychiatry 20, 32–47. 10.1038/mp.2014.163 25486982

[B98] Gray M. A. Harrison N. A. Wiens S. Critchley H. D. (2007). Modulation of emotional appraisal by false physiological feedback during fMRI. PLoS ONE 2:e546. 10.1371/journal.pone.0000546 17579718 PMC1890305

[B99] Gruol D. L. (2015). IL-6 regulation of synaptic function in the CNS. Neuropharmacology 96, 42–54. 10.1016/j.neuropharm.2014.10.023 25445486 PMC4446251

[B100] Gu X. Hof P. R. Friston K. J. Fan J. (2013). Anterior insular cortex and emotional awareness. J. Comp. Neurol. 521, 3371–3388. 10.1002/cne.23368 23749500 PMC3999437

[B101] Guo C. C. Hyett M. P. Nguyen V. T. Parker G. B. Breakspear M. J. (2016). Distinct neurobiological signatures of brain connectivity in depression subtypes during natural viewing of emotionally salient films. Psychol. Med. 46, 1535–1545. 10.1017/S0033291716000179 26888415

[B102] Haider L. Zrzavy T. Hametner S. Hoftberger R. Bagnato F. Grabner G.. (2016). The topograpy of demyelination and neurodegeneration in the multiple sclerosis brain. Brain 139, 807–815. 10.1093/brain/awv398 26912645 PMC4766379

[B103] Hanken K. Eling P. Hildebrandt H. (2014). The representation of inflammatory signals in the brain - a model for subjective fatigue in multiple sclerosis. Front. Neurol. 5:264. 10.3389/fneur.2014.00264 25566171 PMC4263099

[B104] Harrison N. A. Brydon L. Walker C. Gray M. A. Steptoe A. Critchley H. D. (2009a). Inflammation causes mood changes through alterations in subgenual cingulate activity and mesolimbic connectivity. Biol. Psychiatry 66, 407–414. 10.1016/j.biopsych.2009.03.015 19423079 PMC2885494

[B105] Harrison N. A. Brydon L. Walker C. Gray M. A. Steptoe A. Dolan R. J.. (2009b). Neural origins of human sickness in interoceptive responses to inflammation. Biol. Psychiatry 66, 415–422. 10.1016/j.biopsych.2009.03.007 19409533 PMC2885492

[B106] Harrison N. A. Cooper E. Dowell N. G. Keramida G. Voon V. Critchley H. D.. (2015). Quantitative magnetization transfer imaging as a biomarker for effects of systemic inflammation on the brain. Biol. Psychiatry 78, 49–57. 10.1016/j.biopsych.2014.09.023 25526971 PMC4503794

[B107] Hart A. S. Clark J. J. Phillips P. E. (2015). Dynamic shaping of dopamine signals during probabilistic Pavlovian conditioning. Neurobiol. Learn. Mem. 117, 84–92. 10.1016/j.nlm.2014.07.010 25172480 PMC4293327

[B108] Heinzle J. Koopmans P. J. den Ouden H. E. Raman S. Stephan K. E. (2016). A hemodynamic model for layered BOLD signals. Neuroimage 125, 556–570. 10.1016/j.neuroimage.2015.10.025 26484827

[B109] Helmholtz H. (1860/1962). *Handbuch der Physiologischen Optik*, ed Southall J. P. C. New York, NY: Dover.

[B110] Herz D. M. Florin E. Christensen M. S. Reck C. Barbe M. T. Tscheuschler M. K.. (2014). Dopamine replacement modulates oscillatory coupling between premotor and motor cortical areas in Parkinson's disease. Cereb. Cortex 24, 2873–2883. 10.1093/cercor/bht140 23733911 PMC4193459

[B111] Hilgetag C. C. O'Neill M. A. Young M. P. (2000). Hierarchical organization of macaque and cat cortical sensory systems explored with a novel network processor. Philos. Trans. R. Soc. Lond. B. Biol. Sci. 355, 71–89. 10.1098/rstb.2000.0550 10703045 PMC1692720

[B112] Honey C. J. Kotter R. Breakspear M. Sporns O. (2007). Network structure of cerebral cortex shapes functional connectivity on multiple time scales. Proc. Natl. Acad. Sci. U.S.A. 104, 10240–10245. 10.1073/pnas.0701519104 17548818 PMC1891224

[B113] Hsu D. T. Price J. L. (2007). Midline and intralaminar thalamic connections with the orbital and medial prefrontal networks in macaque monkeys. J. Comp. Neurol. 504, 89–111. 10.1002/cne.21440 17626282

[B114] Huitinga I. De Groot C. J. Van der Valk P. Kamphorst W. Tilders F. J. Swaab D. F. (2001). Hypothalamic lesions in multiple sclerosis. J. Neuropathol. Exp. Neurol. 60, 1208–1218. 10.1093/jnen/60.12.1208 11764093

[B115] Huitinga I. Erkut Z. A. van Beurden D. Swaab D. F. (2004). Impaired hypothalamus-pituitary-adrenal axis activity and more severe multiple sclerosis with hypothalamic lesions. Ann. Neurol. 55, 37–45. 10.1002/ana.10766 14705110

[B116] Hurd Y. L. Suzuki M. Sedvall G. C. (2001). D1 and D2 dopamine receptor mRNA expression in whole hemisphere sections of the human brain. J. Chem. Neuroanat. 22, 127–137. 10.1016/S0891-0618(01)00122-3 11470560

[B117] Hurley K. M. Herbert H. Moga M. M. Saper C. B. (1991). Efferent projections of the infralimbic cortex of the rat. J. Comp. Neurol. 308, 249–276. 10.1002/cne.903080210 1716270

[B118] Huys Q. J. Maia T. V. Frank M. J. (2016). Computational psychiatry as a bridge from neuroscience to clinical applications. Nat. Neurosci. 19, 404–413. 10.1038/nn.4238 26906507 PMC5443409

[B119] Hyett M. P. Breakspear M. J. Friston K. J. Guo C. C. Parker G. B. (2015). Disrupted effective connectivity of cortical systems supporting attention and interoception in melancholia. JAMA Psychiatry 72, 350–358. 10.1001/jamapsychiatry.2014.2490 25692565

[B120] Jirsa V. K. Sporns O. Breakspear M. Deco G. McIntosh A. R. (2010). Towards the virtual brain: network modeling of the intact and the damaged brain. Arch. Ital. Biol. 148, 189–205. Available online at: http://www.architalbiol.org/aib/article/view/148189/21175008 21175008

[B121] Kaltsas G. Vgontzas A. Chrousos G. (2010). Fatigue, endocrinopathies, and metabolic disorders. PM R 2, 393–398. 10.1016/j.pmrj.2010.04.011 20656620

[B122] Kass R. E. Steffey D. (1989). Approximate Bayesian inference in conditionally independent hierarchical models (parametric empirical Bayes models). J. Am. Stat. Assoc. 84, 171–726. 10.1080/01621459.1989.10478825

[B123] Keramati M. Gutkin B. (2014). Homeostatic reinforcement learning for integrating reward collection and physiological stability. Elife 3:e04811. 10.7554/eLife.04811 25457346 PMC4270100

[B124] Kersten D. Mamassian P. Yuille A. (2004). Object perception as Bayesian inference. Annu. Rev. Psychol. 55, 271–304. 10.1146/annurev.psych.55.090902.142005 14744217

[B125] Khalsa S. S. Rudrauf D. Damasio A. R. Davidson R. J. Lutz A. Tranel D. (2008). Interoceptive awareness in experienced meditators. Psychophysiology 45, 671–677. 10.1111/j.1469-8986.2008.00666.x 18503485 PMC2637372

[B126] Kluger B. M. Krupp L. B. Enoka R. M. (2013). Fatigue and fatigability in neurologic illnesses: proposal for a unified taxonomy. Neurology 80, 409–416. 10.1212/WNL.0b013e31827f07be 23339207 PMC3589241

[B127] Knill D. Richards W. (1996). Perception as Bayesian Inference. Cambridge: Cambridge University Press.

[B128] Koopmans P. J. Barth M. Norris D. G. (2010). Layer-specific BOLD activation in human V1. Hum. Brain Mapp. 31, 1297–1304. 10.1002/hbm.20936 20082333 PMC6870878

[B129] Körding K. (2007). Decision theory: what “should” the nervous system do? Science 318, 606–610. 10.1126/science.1142998 17962554

[B130] Kroencke D. C. Lynch S. G. Denney D. R. (2000). Fatigue in multiple sclerosis: relationship to depression, disability, and disease pattern. Mult. Scler. 6, 131–136. 10.1177/135245850000600213 10773860

[B131] Larun L. Brurberg K. G. Odgaard-Jensen J. Price J. R. (2016). Exercise therapy for chronic fatigue syndrome. Cochrane Database Syst. Rev. 2:CD003200. 10.1002/14651858.CD003200.pub4 26852189

[B132] Lee T. S. Mumford D. (2003). Hierarchical Bayesian inference in the visual cortex. J. Opt. Soc. Am. A Opt. Image Sci. Vis. 20, 1434–1448. 10.1364/JOSAA.20.001434 12868647

[B133] Lewis D. A. Melchitzky D. S. Sesack S. R. Whitehead R. E. Auh S. Sampson A. (2001). Dopamine transporter immunoreactivity in monkey cerebral cortex: regional, laminar, and ultrastructural localization. J. Comp. Neurol. 432, 119–136. 10.1002/cne.1092 11241381

[B134] Li B. Daunizeau J. Stephan K. E. Penny W. Hu D. Friston K. (2011). Generalised filtering and stochastic DCM for fMRI. Neuroimage 58, 442–457. 10.1016/j.neuroimage.2011.01.085 21310247

[B135] Lieder F. Daunizeau J. Garrido M. I. Friston K. J. Stephan K. E. (2013). Modelling trial-by-trial changes in the mismatch negativity. PLoS Comput. Biol. 9:e1002911. 10.1371/journal.pcbi.1002911 23436989 PMC3578779

[B136] Liu H. Qin W. Li W. Fan L. Wang J. Jiang T.. (2013). Connectivity-based parcellation of the human frontal pole with diffusion tensor imaging. J. Neurosci. 33, 6782–6790. 10.1523/JNEUROSCI.4882-12.2013 23595737 PMC6618893

[B137] Maher-Edwards L. Fernie B. A. Murphy G. Wells A. Spada M. M. (2011). Metacognitions and negative emotions as predictors of symptom severity in chronic fatigue syndrome. J. Psychosom. Res. 70, 311–317. 10.1016/j.jpsychores.2010.09.016 21414450

[B138] Marr D. (1982). Vision: A Computational Investigation into the Human Representation and Processing of Visual Information. New York, NY: Freeman.

[B139] Mathys C. (2016). How could we get nosology from computation?, in Computational Psychiatry: New Perspectives on Mental Illness, eds Reddish D. Gordon J. (Boston, MA: MIT Press).

[B140] Mathys C. Daunizeau J. Friston K. J. Stephan K. E. (2011). A Bayesian foundation for individual learning under uncertainty. Front. Hum. Neurosci. 5:39. 10.3389/fnhum.2011.00039 21629826 PMC3096853

[B141] Mathys C. D. Lomakina E. I. Daunizeau J. Iglesias S. Brodersen K. H. Friston K. J.. (2014). Uncertainty in perception and the Hierarchical Gaussian Filter. Front. Hum. Neurosci. 8:825. 10.3389/fnhum.2014.00825 25477800 PMC4237059

[B142] Mayberg H. S. Liotti M. Brannan S. K. McGinnis S. Mahurin R. K. Jerabek P. A.. (1999). Reciprocal limbic-cortical function and negative mood: converging PET findings in depression and normal sadness. Am. J. Psychiatry 156, 675–682. 10327898 10.1176/ajp.156.5.675

[B143] McCurdy L. Y. Maniscalco B. Metcalfe J. Liu K. Y. de Lange F. P. Lau H. (2013). Anatomical coupling between distinct metacognitive systems for memory and visual perception. J. Neurosci. 33, 1897–1906. 10.1523/JNEUROSCI.1890-12.2013 23365229 PMC4696871

[B144] Mesulam M. M. Mufson E. J. (1982). Insula of the old world monkey. III: efferent cortical output and comments on function. J. Comp. Neurol. 212, 38–52. 10.1002/cne.902120104 7174907

[B145] Miller A. H. Raison C. L. (2016). The role of inflammation in depression: from evolutionary imperative to modern treatment target. Nat. Rev. Immunol. 16, 22–34. 10.1038/nri.2015.5 26711676 PMC5542678

[B146] Modell H. Cliff W. Michael J. McFarland J. Wenderoth M. P. Wright A. (2015). A physiologist's view of homeostasis. Adv. Physiol. Educ. 39, 259–266. 10.1152/advan.00107.2015 26628646 PMC4669363

[B147] Montague P. R. Dolan R. J. Friston K. J. Dayan P. (2012). Computational psychiatry. Trends Cogn. Sci. 16, 72–80. 10.1016/j.tics.2011.11.018 22177032 PMC3556822

[B148] Moran R. J. Stephan K. E. Seidenbecher T. Pape H. C. Dolan R. J. Friston K. J. (2009). Dynamic causal models of steady-state responses. Neuroimage 44, 796–811. 10.1016/j.neuroimage.2008.09.048 19000769 PMC2644453

[B149] Muckli L. De Martino F. Vizioli L. Petro L. S. Smith F. W. Ugurbil K.. (2015). Contextual feedback to superficial layers of V1. Curr. Biol. 25, 2690–2695. 10.1016/j.cub.2015.08.057 26441356 PMC4612466

[B150] Mufson E. J. Mesulam M. M. (1982). Insula of the old world monkey. II: afferent cortical input and comments on the claustrum. J. Comp. Neurol. 212, 23–37. 10.1002/cne.902120103 7174906

[B151] Mumford D. (1992). On the computational architecture of the neocortex. II. The role of cortico-cortical loops. Biol. Cybern. 66, 241–251. 10.1007/BF00198477 1540675

[B152] Nair A. Bonneau R. H. (2006). Stress-induced elevation of glucocorticoids increases microglia proliferation through NMDA receptor activation. J. Neuroimmunol. 171, 72–85. 10.1016/j.jneuroim.2005.09.012 16278020

[B153] Nassi J. J. Lomber S. G. Born R. T. (2013). Corticocortical feedback contributes to surround suppression in V1 of the alert primate. J. Neurosci. 33, 8504–8517. 10.1523/JNEUROSCI.5124-12.2013 23658187 PMC3690087

[B154] Olman C. A. Harel N. Feinberg D. A. He S. Zhang P. Ugurbil K.. (2012). Layer-specific fMRI reflects different neuronal computations at different depths in human V1. PLoS ONE 7:e32536. 10.1371/journal.pone.0032536 22448223 PMC3308958

[B155] Parker G. Paterson A. (2014). Melancholia: definition and management. Curr. Opin. Psychiatry 27, 1–6. 10.1097/YCO.0000000000000024 24270479

[B156] Patejdl R. Penner I. K. Noack T. K. Zettl U. K. (2016). Multiple sclerosis and fatigue: a review on the contribution of inflammation and immune-mediated neurodegeneration. Autoimmun. Rev. 15, 210–220. 10.1016/j.autrev.2015.11.005 26589194

[B157] Paulus M. P. Flagan T. Simmons A. N. Gillis K. Kotturi S. Thom N.. (2012). Subjecting elite athletes to inspiratory breathing load reveals behavioral and neural signatures of optimal performers in extreme environments. PLoS ONE 7:e29394. 10.1371/journal.pone.0029394 22276111 PMC3261851

[B158] Paulus M. P. Stein M. B. (2006). An insular view of anxiety. Biol. Psychiatry 60, 383–387. 10.1016/j.biopsych.2006.03.042 16780813

[B159] Penny W. D. (2012). Comparing dynamic causal models using AIC, BIC and free energy. Neuroimage 59, 319–330. 10.1016/j.neuroimage.2011.07.039 21864690 PMC3200437

[B160] Penny W. Stephan K. E. (2014). A dynamic Bayesian model of homeostatic control. Lecture Notes Comput. Sci. 8779, 60–69. 10.1007/978-3-319-11298-5_7

[B161] Petzschner F. H. Glasauer S. Stephan K. E. (2015). A Bayesian perspective on magnitude estimation. Trends Cogn. Sci. 19, 285–293. 10.1016/j.tics.2015.03.002 25843543

[B162] Pezzulo G. Rigoli F. Friston K. (2015). Active Inference, homeostatic regulation and adaptive behavioural control. Prog. Neurobiol. 134, 17–35. 10.1016/j.pneurobio.2015.09.001 26365173 PMC4779150

[B163] Pittion-Vouyovitch S. Debouverie M. Guillemin F. Vandenberghe N. Anxionnat R. Vespignani H. (2006). Fatigue in multiple sclerosis is related to disability, depression and quality of life. J. Neurol. Sci. 243, 39–45. 10.1016/j.jns.2005.11.025 16434057

[B164] Popescu V. Schoonheim M. M. Versteeg A. Chaturvedi N. Jonker M. de Menezes R. X.. (2016). Grey matter atrophy in multiple sclerosis: clinical interpretation depends on choice of analysis method. PLoS One 11:e0143942. 10.1371/journal.pone.0143942 26745873 PMC4706325

[B165] Powers W. T. (1973). Feedback: beyond behaviorism. Science 179, 351–356. 10.1126/science.179.4071.351 4682961

[B166] Powers W. T. (1978). Quantitative analysis of purposive systems: some spadework at the foundations of scientific psychology. Psychol. Rev. 85, 417–435. 10.1037/0033-295X.85.5.417

[B167] Raman S. Deserno L. Schlagenhauf F. Stephan K. E. (2016). A hierarchical model for integrating unsupervised generative embedding and empirical Bayes. J. Neurosci. Methods 269, 6–20. 10.1016/j.jneumeth.2016.04.022 27141854

[B168] Rao R. P. Ballard D. H. (1999). Predictive coding in the visual cortex: a functional interpretation of some extra-classical receptive-field effects. Nat. Neurosci. 2, 79–87. 10.1038/4580 10195184

[B169] Richards J. von Glasersfeld E. (1979). The control of perception and the construction of reality: epistemological aspects of the feedback-control system. Dialectica 33, 37–58. 10.1111/j.1746-8361.1979.tb01519.x

[B170] Rosenbaum M. Hadari D. (1985). Personal efficacy, external locus of control, and perceived contingency of parental reinforcement among depressed, paranoid, and normal subjects. J. Pers. Soc. Psychol. 49, 539–547. 10.1037/0022-3514.49.2.539 4032231

[B171] Saper C. B. (2002). The central autonomic nervous system: conscious visceral perception and autonomic pattern generation. Annu. Rev. Neurosci. 25, 433–469. 10.1146/annurev.neuro.25.032502.111311 12052916

[B172] Sapolsky R. M. (2015). Stress and the brain: individual variability and the inverted-U. Nat. Neurosci. 18, 1344–1346. 10.1038/nn.4109 26404708

[B173] Schlagenhauf F. Huys Q. J. Deserno L. Rapp M. A. Beck A. Heinze H. J.. (2014). Striatal dysfunction during reversal learning in unmedicated schizophrenia patients. Neuroimage 89, 171–180. 10.1016/j.neuroimage.2013.11.034 24291614 PMC3991847

[B174] Schmidt A. Diaconescu A. O. Kometer M. Friston K. J. Stephan K. E. Vollenweider F. X. (2013). Modeling ketamine effects on synaptic plasticity during the mismatch negativity. Cereb. Cortex 23, 2394–2406. 10.1093/cercor/bhs238 22875863 PMC3767962

[B175] Schnyer D. M. Verfaellie M. Alexander M. P. LaFleche G. Nicholls L. Kaszniak A. W. (2004). A role for right medial prefontal cortex in accurate feeling-of-knowing judgements: evidence from patients with lesions to frontal cortex. Neuropsychologia 42, 957–966. 10.1016/j.neuropsychologia.2003.11.020 14998710

[B176] Schwartenbeck P. FitzGerald T. H. Mathys C. Dolan R. Friston K. (2015a). The dopaminergic midbrain encodes the expected certainty about desired outcomes. Cereb. Cortex 25, 3434–3445. 10.1093/cercor/bhu159 25056572 PMC4585497

[B177] Schwartenbeck P. FitzGerald T. H. Mathys C. Dolan R. Wurst F. Kronbichler M.. (2015b). Optimal inference with suboptimal models: addiction and active Bayesian inference. Med. Hypotheses 84, 109–117. 10.1016/j.mehy.2014.12.007 25561321 PMC4312353

[B178] Seth A. K. (2013). Interoceptive inference, emotion, and the embodied self. Trends Cogn. Sci. 17, 565–573. 10.1016/j.tics.2013.09.007 24126130

[B179] Seth A. K. (2015a). The cybernetic Bayesian brain: from interoceptive inference to sensorimotor contingencies, in Open MIND, eds Metzinger T. Windt J. (Frankfurt: MIND Group), 1–24. 10.15502/9783958570108

[B180] Seth A. K. (2015b). Inference to the best prediction, in Open MIND, eds Metzinger T. Windt J. (Frankfurt: MIND Group), 1–8. 10.15502/9783958570986

[B181] Seth A. K. (2015c). Presence, objecthood, and the phenomenology of predictive perception. Cogn. Neurosci. 6, 111–117. 10.1080/17588928.2015.1026888 25849361

[B182] Seth A. K. Chorley P. Barnett L. C. (2013). Granger causality analysis of fMRI BOLD signals is invariant to hemodynamic convolution but not downsampling. Neuroimage 65, 540–555. 10.1016/j.neuroimage.2012.09.049 23036449

[B183] Seth A. K. Suzuki K. Critchley H. D. (2011). An interoceptive predictive coding model of conscious presence. Front. Psychol. 2:395. 10.3389/fpsyg.2011.00395 22291673 PMC3254200

[B184] Shattuck E. C. Muehlenbein M. P. (2015). Human sickness behavior: ultimate and proximate explanations. Am. J. Phys. Anthropol. 157, 1–18. 10.1002/ajpa.22698 25639499

[B185] Shimamura A. P. Squire L. R. (1986). Memory and metamemory: a study of the feeling-of-knowing phenomenon in amnesic patients. J. Exp. Psychol. Learn. Mem. Cogn. 12, 452–460. 10.1037/0278-7393.12.3.452 2942629

[B186] Skapinakis P. Lewis G. Mavreas V. (2004). Temporal relations between unexplained fatigue and depression: longitudinal data from an international study in primary care. Psychosom. Med. 66, 330–335. 10.1097/01.psy.0000124757.10167.b1 15184691

[B187] Srinivasan M. V. Laughlin S. B. Dubs A. (1982). Predictive coding: a fresh view of inhibition in the retina. Proc. R. Soc. Lond. B Biol. Sci. 216, 427–459. 10.1098/rspb.1982.0085 6129637

[B188] Stephan K. E. Bach D. R. Fletcher P. C. Flint J. Frank M. J. Friston K. J.. (2016a). Charting the landscape of priority problems in psychiatry, part 1: classification and diagnosis. Lancet Psychiatry 3, 77–83. 10.1016/S2215-0366(15)00361-2 26573970

[B189] Stephan K. E. Diaconescu A. O. Iglesias S. (2016b). Bayesian inference, dysconnectivity and neuromodulation in schizophrenia. Brain 139, 1874–1876. 10.1093/brain/aww120 27343221

[B190] Stephan K. E. Iglesias S. Heinzle J. Diaconescu A. O. (2015). Translational perspectives for computational neuroimaging. Neuron 87, 716–732. 10.1016/j.neuron.2015.07.008 26291157

[B191] Stephan K. E. Kasper L. Harrison L. M. Daunizeau J. den Ouden H. E. Breakspear M.. (2008). Nonlinear dynamic causal models for fMRI. Neuroimage 42, 649–662. 10.1016/j.neuroimage.2008.04.262 18565765 PMC2636907

[B192] Stephan K. E. Mathys C. (2014). Computational approaches to psychiatry. Curr. Opin. Neurobiol. 25, 85–92. 10.1016/j.conb.2013.12.007 24709605

[B193] Stephan K. E. Penny W. D. Daunizeau J. Moran R. J. Friston K. J. (2009). Bayesian model selection for group studies. Neuroimage 46, 1004–1017. 10.1016/j.neuroimage.2009.03.025 19306932 PMC2703732

[B194] Sterling P. (2012). Allostasis: a model of predictive regulation. Physiol. Behav. 106, 5–15. 10.1016/j.physbeh.2011.06.004 21684297

[B195] Sterling P. (2014). Homeostasis vs. allostasis: implications for brain function and mental disorders. JAMA Psychiatry 71, 1192–1193. 10.1001/jamapsychiatry.2014.1043 25103620

[B196] Stewart J. M. (2000). Autonomic nervous system dysfunction in adolescents with postural orthostatic tachycardia syndrome and chronic fatigue syndrome is characterized by attenuated vagal baroreflex and potentiated sympathetic vasomotion. Pediatr. Res. 48, 218–226. 10.1203/00006450-200008000-00016 10926298

[B197] Stuke K. Flachenecker P. Zettl U. K. Elias W. G. Freidel M. Haas J.. (2009). Symptomatology of MS: results from the German MS Registry. J. Neurol. 256, 1932–1935. 10.1007/s00415-009-5257-5 19629565

[B198] Tomassini A. Ruge D. Galea J. M. Penny W. Bestmann S. (2016). The role of dopamine in temporal uncertainty. J. Cogn. Neurosci. 28, 96–110. 10.1162/jocn_a_00880 26401816

[B199] Tsigos C. Chrousos G. P. (2002). Hypothalamic-pituitary-adrenal axis, neuroendocrine factors and stress. J. Psychosom. Res. 53, 865–871. 10.1016/S0022-3999(02)00429-4 12377295

[B200] Vetter P. Grosbras M. H. Muckli L. (2015). TMS over V5 disrupts motion prediction. Cereb. Cortex 25, 1052–1059. 10.1093/cercor/bht297 24152544 PMC4380002

[B201] Vezzani A. Viviani B. (2015). Neuromodulatory properties of inflammatory cytokines and their impact on neuronal excitability. Neuropharmacology 96, 70–82. 10.1016/j.neuropharm.2014.10.027 25445483

[B202] Vinckier F. Gaillard R. Palminteri S. Rigoux L. Salvador A. Fornito A.. (2016). Confidence and psychosis: a neuro-computational account of contingency learning disruption by NMDA blockade. Mol. Psychiatry 21, 946–955. 10.1038/mp.2015.73 26055423 PMC5414075

[B203] Vogt B. A. (2005). Pain and emotion interactions in subregions of the cingulate gyrus. Nat. Rev. Neurosci. 6, 533–544. 10.1038/nrn1704 15995724 PMC2659949

[B204] Vogt B. A. Pandya D. N. (1987). Cingulate cortex of the rhesus monkey: II. Cortical afferents. J. Comp. Neurol. 262, 271–289. 10.1002/cne.902620208 3624555

[B205] Von Bertalanffy L. (1969). General System Theory. New York, NY: George Braziller.

[B206] von Foerster H. (2003). Understanding Understanding: Essays on Cybernetics and Cognition. New York, NY: Springer.

[B207] Wacongne C. Changeux J. P. Dehaene S. (2012). A neuronal model of predictive coding accounting for the mismatch negativity. J. Neurosci. 32, 3665–3678. 10.1523/JNEUROSCI.5003-11.2012 22423089 PMC6703454

[B208] Wessely S. (2001). Chronic fatigue: symptom and syndrome. Ann. Intern. Med. 134, 838–843. 10.7326/0003-4819-134-9_Part_2-200105011-00007 11346319

[B209] Wessely S. Chalder T. Hirsch S. Wallace P. Wright D. (1996). Psychological symptoms, somatic symptoms, and psychiatric disorder in chronic fatigue and chronic fatigue syndrome: a prospective study in the primary care setting. Am. J. Psychiatry 153, 1050–1059. 10.1176/ajp.153.8.1050 8678174

[B210] Wiener N. (1948). Cybernetics. New York, NY: Wiley.

[B211] Woods H. A. Wilson J. K. (2013). An information hypothesis for the evolution of homeostasis. Trends Ecol. Evol. 28, 283–289. 10.1016/j.tree.2012.10.021 23182696

[B212] Woolrich M. W. Stephan K. E. (2013). Biophysical network models and the human connectome. Neuroimage 80, 330–338. 10.1016/j.neuroimage.2013.03.059 23571421

[B213] Zacharopoulos G. Binetti N. Walsh V. Kanai R. (2014). The effect of self-efficacy on visual discrimination sensitivity. PLoS ONE 9:e109392. 10.1371/journal.pone.0109392 25295529 PMC4190082

